# Dimensional Engineering of Carbon‐Based Hybrids: From 1D Conductive Networks to 3D Porous Architectures for Next‐Generation Microwave Absorption

**DOI:** 10.1002/advs.77294

**Published:** 2026-08-20

**Authors:** Chuanyu Liu, Xiaoxin Guo, Xunlong Zhang, Ruiling Sun, Xingyu Zhang, Can Ge, Xuhong Yang, Yuqing Liu

**Affiliations:** ^1^ College of Textile and Clothing Engineering Soochow University Suzhou China; ^2^ National Engineering Laboratory for Modern Silk College of Textile and Clothing Engineering Soochow University Suzhou China; ^3^ College of Polymer Science and Engineering Sichuan University Chengdu China; ^4^ Department of Electronic Engineering and Shun Hing Institute of Advanced Engineering The Chinese University of Hong Kong Hong Kong China

**Keywords:** carbon nanofiber (CNF) composites, dimensional architecture, EWAMs, MOF‐derived materials, MXenes, structure–property relationships

## Abstract

The escalating electromagnetic interference (EMI) from GHz to THz frequencies necessitates advanced electromagnetic wave absorbing materials (EWAMs). This review explores dimensional architecture engineering to overcome the trade‐off between impedance matching and attenuation. We analyze 1D carbon nanofibers for conductivity, two‐dimensional MXenes for interfacial polarization, and 3D MOF‐derived frameworks for multiple scattering. Emphasis is placed on multidimensional hybrids (e.g., 1D/2D, 2D/3D) that synergistically enhance performance beyond single‐dimensional limits. Critical challenges like environmental stability, scalability, and standardized testing are addressed. Finally, we propose future directions including dynamic tunability, multifunctional integration, and AI‐driven design, offering a robust framework for next‐generation intelligent EWAMs achieving thin, lightweight, broadband, and strong absorption.

## Graphical abstract

This review explores dimensional architecture engineering to overcome the impedance‐attenuation trade‐off in electromagnetic wave absorbing materials. By analyzing 1D to 3D structures and their multidimensional hybrids, we highlight synergistic mechanisms for enhanced performance. Future directions in AI‐driven design and intelligent materials are proposed for next‐generation broadband absorption.

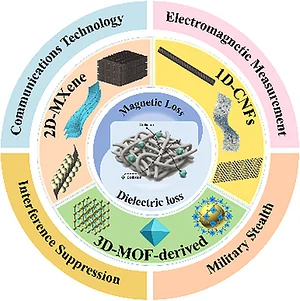

## 1 Introduction

The exponential proliferation of wireless communication technologies and high‐frequency electronic devices has precipitated a critical surge in electromagnetic interference (EMI) and radiation pollution, posing severe threats to both information security and human health [1, 2, 3]. Consequently, the development of high‐performance electromagnetic wave absorption (EMWA) materials has become a strategic imperative in fields ranging from aerospace stealth to civilian electronics protection. Traditional absorbers, such as ferrites and conductive polymers, often suffer from inherent limitations including high density, narrow bandwidth, and single‐loss mechanisms, failing to meet the increasingly stringent demands for “thin, lightweight, wide‐band, and strong” (TLWS) performance [4, 5, 6]. Among various candidates, 1D carbon nanofibers (CNFs) have garnered immense attention due to their exceptional electrical conductivity, chemical stability, and high aspect ratio, which facilitate the formation of conductive networks [7, 8, 9] Supporting Information.

However, relying solely on the intrinsic properties of isolated 1D CNFs encounters a fundamental bottleneck: the dimensional paradox. While 1D topology favors electron transport, random accumulation of nanofibers often leads to dense packing, severe impedance mismatch, and limited internal reflection surfaces. This results in most incident waves being reflected rather than absorbed [10, 11, 12, 13]. Furthermore, pure 1D assemblies lack sufficient polarization centers and structural robustness for practical applications. It has become evident that unlocking the full potential of CNFs requires transcending their native 1D form through dimensional engineering‐evolving from simple 1D fillers to complex 3D hierarchical architectures.

A wealth of previous reviews and experimental works have investigated microwave absorption in carbon/MXene hybrids, aerogels and hollow microspheres [14, 15, 16, 17]. These early efforts primarily focus on synthetic strategies, structural design and intrinsic electromagnetic performances of monocomponent carbon or MXene materials. Regrettably, microscopic doping, mesoscopic confinement and macroscopic assembly are commonly studied independently, without forming a unified theoretical system for structural evolution. A comprehensive overview covering the transition from 1D to 3D geometries remains lacking to this day.

Recent state‐of‐the‐art breakthroughs have also been documented [18, 19, 20], which have greatly advanced the development of carbon‐based microwave absorbers. Yet these works still lack systematic analysis on the structural merits and synergistic effects of multidimensional composite systems. Accordingly, it is critical to reveal how multi‐scale dimensional engineering optimizes the balance between electromagnetic attenuation and impedance matching.

Against this research background, recent breakthroughs demonstrate that the key to high‐performance EMWA lies in a systematic dimensional evolution strategy. Recent breakthroughs demonstrate that the key to high‐performance EMWA lies in a systematic dimensional evolution strategy. Retaining the 1D Advantage: Utilizing CNFs as conductive backbones to ensure efficient charge transport and mechanical flexibility [21, 22, 23]. Introducing 2D Interfaces: Hybridizing 1D CNFs with 2D materials (e.g., MXenes, graphene) creates abundant heterogeneous interfaces. Here, CNFs act as “spacers” to prevent 2D sheet restacking, while the 2D sheets provide massive surface areas for interfacial polarization and multiple scattering [24, 25, 26]. Constructing 3D Architectures: Assembling these 1D/2D building blocks into macroscopic 3D porous networks (e.g., MOF derivatives, aerogels, foams). This dimensional leap transforms the material from a dense bulk into a lightweight framework, promoting prolonged wave propagation paths via internal multiple reflections and significantly improving impedance matching.

Despite these advances, existing reviews often treat microscopic doping, mesoscopic confinement, and macroscopic assembly as isolated strategies. A comprehensive perspective that systematically connects these approaches through the lens of “1D‐to‐3D Dimensional Evolution” is still lacking. There is an urgent need to clarify how manipulating dimensionality at each scale synergistically optimizes the balance between attenuation capability and impedance matching.

To bridge this gap, we propose a unified framework centered on dimensional synergy, categorizing recent breakthroughs into three evolutionary stages:

1. 1D Intrinsic Modulation: Enhancing dielectric loss via atomic‐level defect and doping engineering within the nanofiber itself.
2. 1D/2D Dimensional Hybridization: Optimizing interfacial polarization and magnetic‐dielectric balance through spatial confinement and heterostructure construction.
3. 3D Macroscopic Assembly: Designing porous materials to maximize multiple scattering and broaden the effective absorption bandwidth.

**FIGURE 1:**
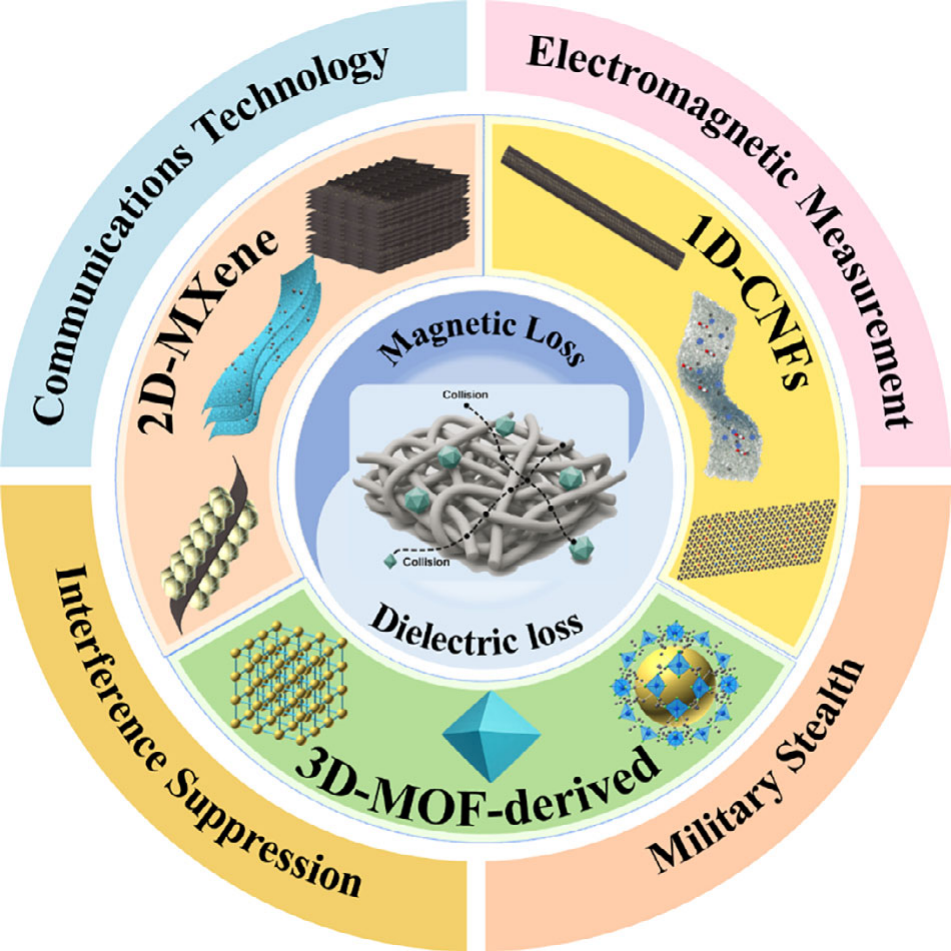
Various composites to enhance the electromagnetic wave absorption.

Furthermore, we critically examine how this 1D‐to‐3D architectural evolution extends the functionality of CNFs beyond EMWA to EMI shielding, flexible sensing, and intelligent thermal management. By correlating specific dimensional features with performance metrics, this review aims to provide actionable guidelines for designing next‐generation smart materials and outlines a roadmap for their transition from laboratory concepts to industrial applications (Figure 1).

## 2 Mechanisms and Design Principles of Electromagnetic Wave Absorbers

Energy dissipation of incident electromagnetic waves within absorbers is dominated by dielectric loss and magnetic loss [27]. Efficient conversion of electromagnetic energy into thermal energy is realized via synergistic modulation of the micro/nanostructure and electromagnetic field responses of materials. As the fundamental electromagnetic parameters, the complex permittivity (ε_r_) and complex permeability (µ_r_) govern the interaction between electromagnetic waves and absorbing materials [28, 29]. Rational structural design enables the effective regulation of both dielectric and magnetic loss mechanisms. Such designs help balance the inherent trade‐off between impedance matching and attenuation capability.

### 2.1 Dielectric Loss

**FIGURE 2:**
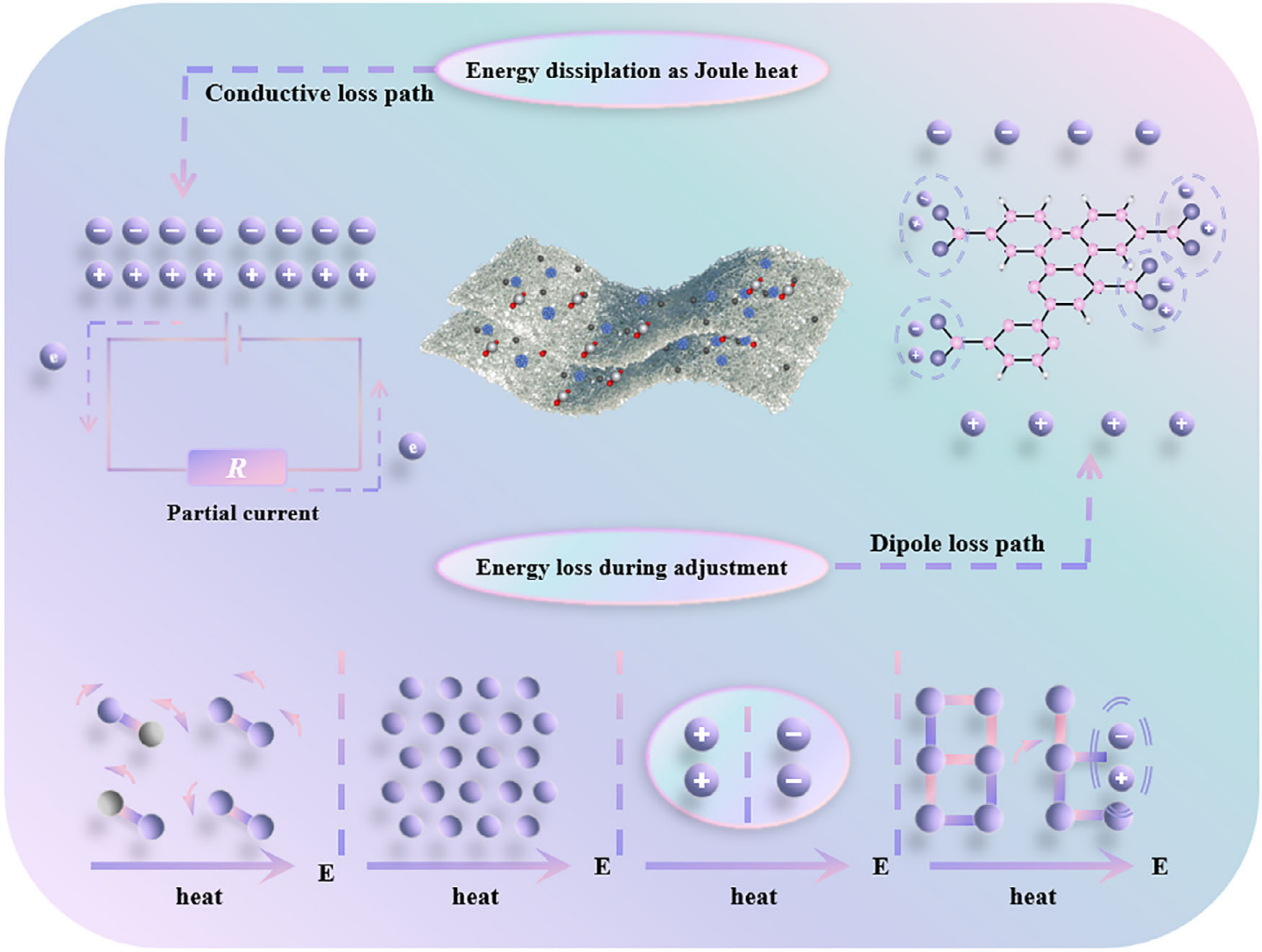
Dielectric loss.

Dielectric loss is considered the primary energy dissipation mechanism in carbon‐based and MXene‐based absorbers. Dielectric loss is governed by conductive loss and polarization relaxation loss (Figure 2) [30]. These processes govern the conversion of electromagnetic energy into thermal energy and can be interpreted within the framework of Debye relaxation theory [31, 32].

#### 2.1.1 Conductive Loss

This mechanism originates from the directional migration of free charge carriers within conductive networks under an alternating electric field. The free carriers primarily include electrons and holes. Such migration is driven by alternating external electric fields. During migration, charge carriers undergo frequent scattering events with lattice atoms, defects, and interfaces. These scattering processes result in irreversible energy dissipation in the form of Joule heating. Complex conductivity is selected as the core physical parameter to describe conductive loss. This parameter is expressed in Equation 1 [33]:

(1)

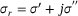

where the fundamental part *σ'* characterizes the ohmic loss, which decreases with increasing frequency, and the imaginary part *σ''* reflects the phase lag of the carriers in response to the electric field, resulting in inductive losses. It is essential to recognize that when conductivity is excessively high, the strong skin effect prevails, resulting in the predominant reflection of energy within the material's surface layer [34]. This results in a substantial impedance mismatch. Consequently, the microwave absorption performance of highly conductive materials is often severely limited. Conductivity must be carefully regulated through composition and structural engineering.

#### 2.1.2 Polarization Loss

Polarization relaxation loss constitutes another major contribution to dielectric loss. Internal dipoles cannot instantaneously follow the rapid oscillation of an alternating electric field. The resulting phase lag between dipole orientation and field variation leads to energy dissipation [35]. Polarization loss in practical absorbing materials is divided into dipole relaxation and interfacial polarization. The magnitude of polarization loss strongly depends on material composition, defect density, and interfacial architecture.

Intrinsic polar functional groups, heteroatom dopants, and structural defects can all act as dipole polarization centers. These defects and functional groups serve as dipole polarization centers under an applied electric field. These dipoles undergo orientation polarization in response to the external field. This is much more professional. The polarization response lags behind field changes to the maximum extent at a specific external field frequency. This specific frequency is defined as the characteristic relaxation frequency *f_c_* of dipoles. A maximum dielectric‐loss response is typically observed near this characteristic relaxation frequency (Equation 2),

(2) $$f_{c}=\frac{1}{2\pi \tau }$$

defined by the relaxation time τ, the polarization response lags maximally behind the field variation. This resonance‐like condition maximizes the phase difference, leading to peak dipole loss.

In heterogeneous systems, discrepancies in electrical properties (conductivity and permittivity) across distinct phases cause charge accumulation at interfaces. Under an alternating field, the continuous buildup and relaxation of these space charges generate significant interfacial polarization(Maxwell‐Wagner‐Sillars Effect) loss [36]. Heterogeneous interfaces are highly effective in enhancing interfacial polarization loss. Porous structural engineering further promotes interfacial polarization by increasing the density of heterogeneous interfaces and charge‐trapping sites.

### 2.2 Magnetic Loss

**FIGURE 3:**
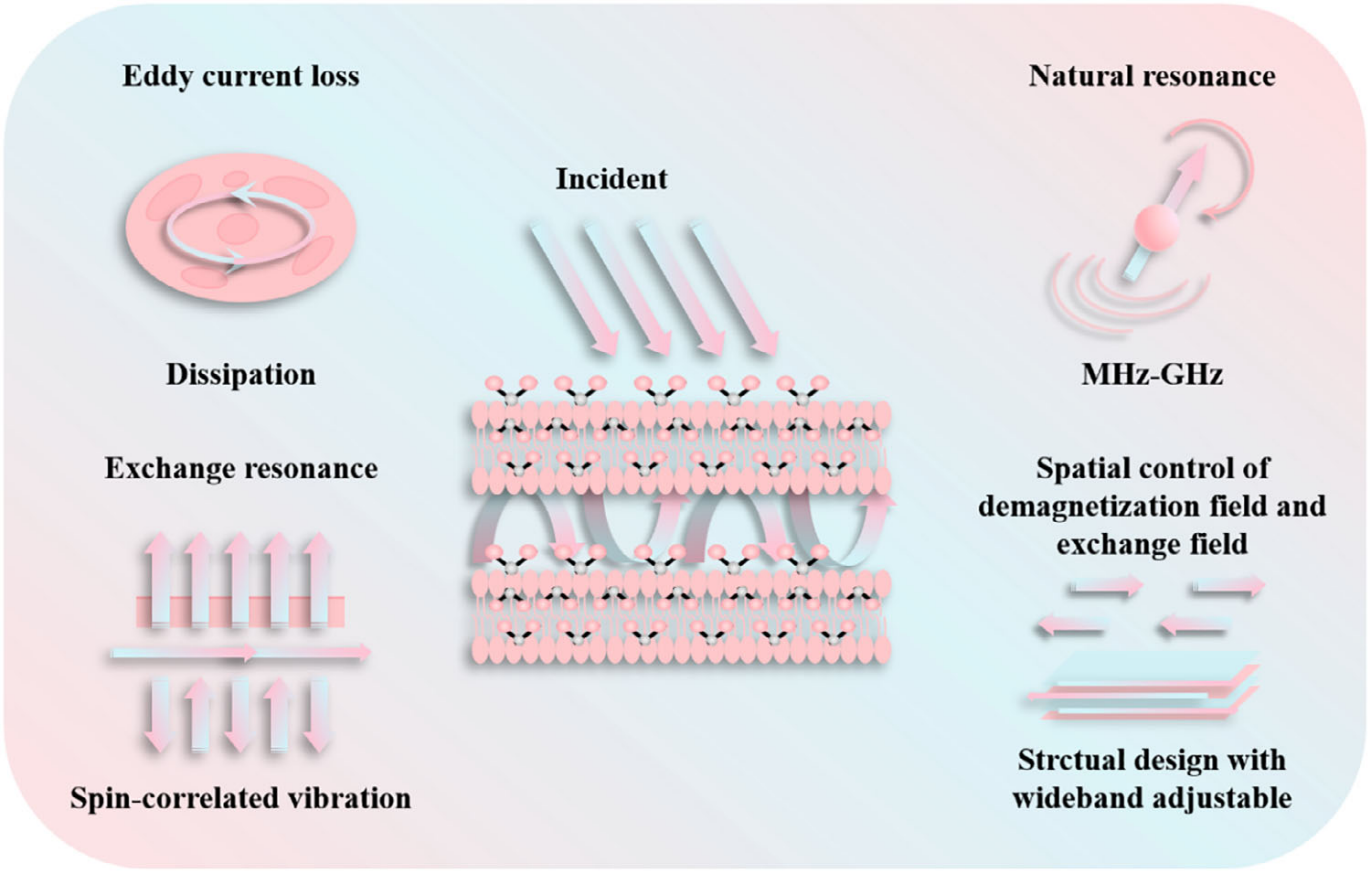
Magnetic loss mechanism.

Magnetic loss is the primary attenuation mechanism in magnetic microwave absorbers. Magnetic loss originates from the dynamic response of magnetic moments to high‐frequency alternating electromagnetic fields as shown in Figure 3. The energy dissipation intensity of magnetic loss is quantitatively characterized by the imaginary part of complex permeability(µ*''*). Several physical mechanisms contribute to magnetic loss. These mechanisms include natural resonance, exchange resonance, eddy‐current loss, hysteresis loss, and domain‐wall motion [37, 38]. Eddy current loss is classified as a typical non‐resonant magnetic loss mechanism. Induced vortex currents are generated inside conductive magnetic materials under time‐varying magnetic fields. Energy is dissipated in the form of Joule heat through these vortex currents. The eddy current coefficient *C*_0_ is applied to identify the contribution of eddy current loss. When *C*_0_ remains approximately constant over a frequency range, eddy‐current loss is generally considered the dominant magnetic‐loss mechanism. Resonance mechanisms are regarded as the dominant factor when *C*_0_ changes with frequency [39, 40].

Among these, magnetic resonance‐comprising natural and exchange resonance represents the most efficient loss channel. Natural resonance stems from the precession of magnetic moments around the magnetocrystalline anisotropy field. When the incident wave frequency matches this intrinsic precession frequency (typically MHz to GHz), maximal energy absorption occurs. Exchange resonance originates from exchange interactions between neighboring magnetic moments and generally appears in the higher‐frequency, depending on the magnetic anisotropy and exchange stiffness of the material. The frequencies of these resonances are primarily determined by magnetic anisotropy, saturation magnetization, and exchange interactions. Conversely, hysteresis loss and domain‐wall resonance generally occur at lower frequencies (< GHz) or under strong static fields and are often negligible in high‐frequency microwave applications [41].

While surface geometry significantly influences magnetic interactions, the precise structure‐loss relationship remains incompletely understood, with current research largely confined to optimizing resonance and eddy current contributions. Future advances may be achieved through the precise regulation of demagnetizing fields, exchange interactions, and spin dynamics via advanced structural engineering [42]. Strategies such as suppressing demagnetizing field inhomogeneity can broaden the magnetic loss peak across wide frequency bands, while spatially separated magnetic texturing can extend topological excitation frequencies beyond the GHz range while mitigating stray fields [43]. Furthermore, elucidating the mapping laws between spin dynamics and loss types at laminar interfaces promises to transcend the traditional eddy‐current‐resonance framework, offering deeper insights into magnetic loss mechanisms [44, 45].

### 2.3 Impedance Matching and Multilayer Structure Design

The reflection behavior of electromagnetic waves at a material interface is determined by the match between the dielectric characteristic impedance (*Z*_in_) and the free space impedance (*Z*_0_). The impedance mismatch phenomenon causes the incident wave to be reflected at the interface, reducing the effective absorption of electromagnetic waves by the material. When the impedance of the sample matches that of air, the electromagnetic wave can completely penetrate the sample; thus, impedance is a prerequisite for excellent electromagnetic wave‐absorbing materials. The synergistic tuning of the real and imaginary parts of the complex permittivity and complex permeability is a core parameter for realizing broadband impedance matching.

The impedance‐matching frequency band of a single‐layer wave‐absorbing material is strongly correlated with its thickness and is also influenced by the complex permittivity and complex permeability. This is known as the quarter‐wavelength principle (Equation 3):

(3) $$d=\frac{n\lambda }{4}=\frac{nc}{4f\sqrt{\left|\mu_{r}\left|\varepsilon_{r}\right|\right|}}\;\left(n=1,3,5\right)$$

In this equation, *d* denotes the thickness of the maximum *R*_L_ value, *ƒ* is the frequency corresponding to the maximum *R*_L_ value, and *λ*_0_ is the free space wavelength. To achieve impedance matching and maximum reflection loss, the quarter‐wavelength matching condition must be satisfied. However, this condition is only valid at a specific frequency, i.e., near the resonant frequency ƒ_c_. This results in the adequate bandwidth being limited to a relatively narrow frequency band. Furthermore, the penetration depth of electromagnetic waves at high frequencies will decrease due to the skin effect. A large number of electromagnetic waves will be reflected near the surface layer. Conversely, a significant increase in thickness is required at low frequencies to maintain the wave absorption efficiency. Consequently, it is challenging to simultaneously meet the demand for low reflection and high absorption over a broad frequency range, thereby impeding the pursuit of lightweight engineering.

Constructing multilayer architectures provides an effective strategy for enhancing microwave absorption without increasing the overall material loading [46]. By stacking layers with disparate electromagnetic parameters, a continuous impedance gradient can be established from free space to the high‐loss interior, effectively circumventing bandwidth limitations. The surface layer modulates the reflection *Φ* (Equation 4):

(4) $$\Phi =\frac{4\pi }{\lambda_{0}}\;\sqrt{\mu \varepsilon d}=\left(2n+1\right)\;\pi ,n=0,1,2,3\text{…}$$

to achieve coherent cancellation of the incident and reflected waves. The middle layer employs a gradient distribution of dielectric constants to form a continuous impedance transition (the normalized impedance of the nth layer should satisfy *Z_n_* (Equation 5):

(5) $$Z_{n}=Z_{0}\;\prod_{i=1}^{n}\sqrt{\frac{\mu^{\left(i\right)}}{\varepsilon^{\left(i\right)}}}$$

**FIGURE 4:**
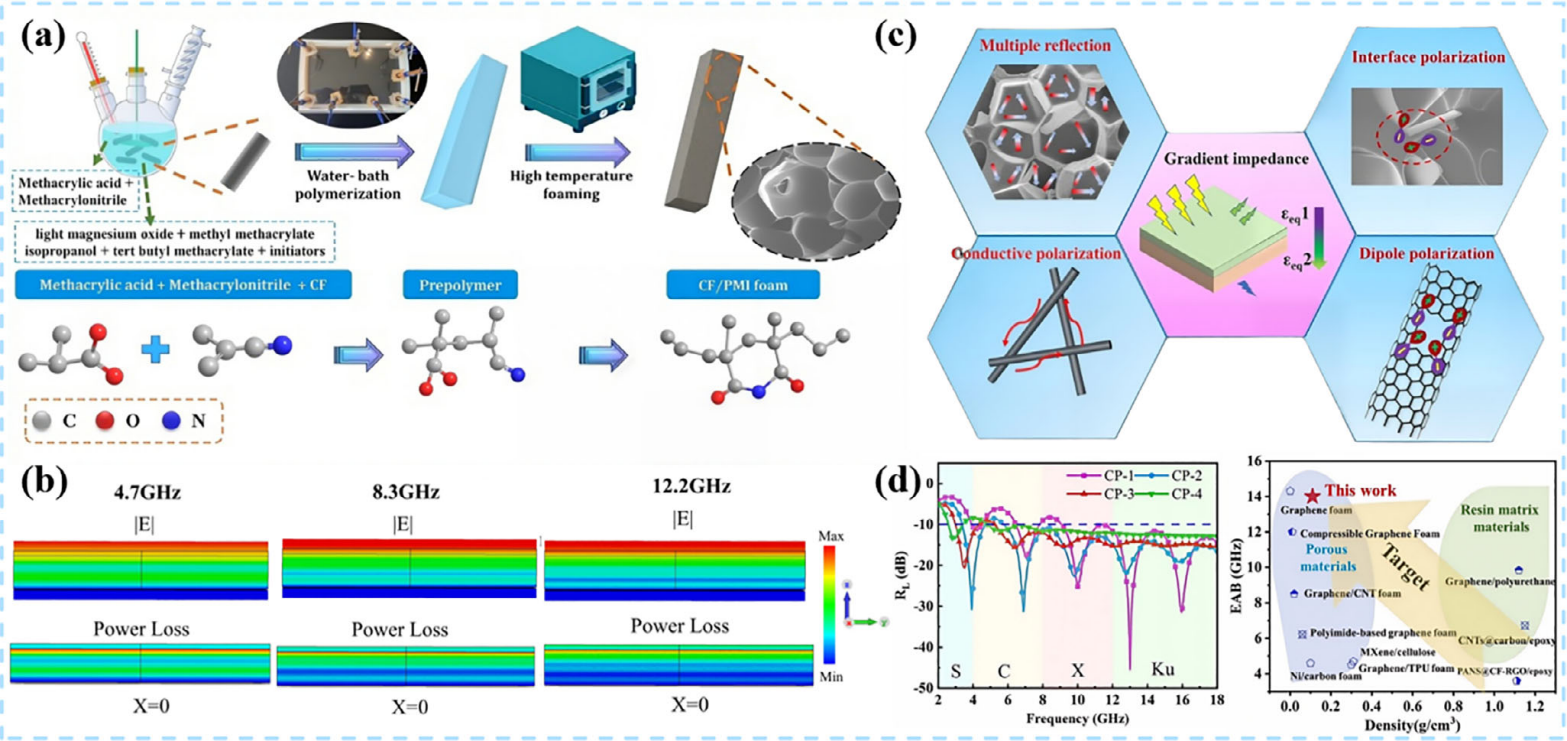
(a) Schematic illustration of the synthesis process for CP composites, (b) simulation of electric field intensity and power loss at 4.7, 8.3, and 12.2 GHz, (c) schematic diagram of the absorption mechanism of CP composites, (d) *R*_L_ curves of CP composites with a thickness of 4 cm and comparison of MA performance with other previously reported absorbers [47] Copyright 2022, Springer.

Conversely, the bottom layer utilizes dielectric relaxation and magnetic resonance mechanisms to dissipate electromagnetic energy, as detailed in the preceding section. Broadband absorption arises from the combined effects of impedance‐gradient matching, multiple internal reflections, and phase cancellation among reflected waves. Jiao et al. [47] demonstrated high‐temperature absorption by constructing multi‐layer composite porous structures (Figure 4a). In this design, the exterior layer possesses impedance properties analogous to air, facilitating wave entry, while the interior layer provides superior attenuation. This configuration allows electromagnetic waves to penetrate deeply and convert efficiently into heat. Additionally, the increased interfacial area in multi‐layer architectures promotes space charge accumulation (Figure 4b) and introduces interfacial defects that enhance dipole polarization (Figure 4c,d), collectively boosting loss capability. Based on equivalent transmission line theory, such multi‐layer systems can be modeled as series impedance networks. By strategically optimizing the electromagnetic parameters and thickness of each layer, the total input impedance and attenuation efficiency can be precisely tailored to achieve broadband, high‐performance absorption.

### 2.4 Porous Structure and Interfacial Reflection Modulation

**FIGURE 5:**
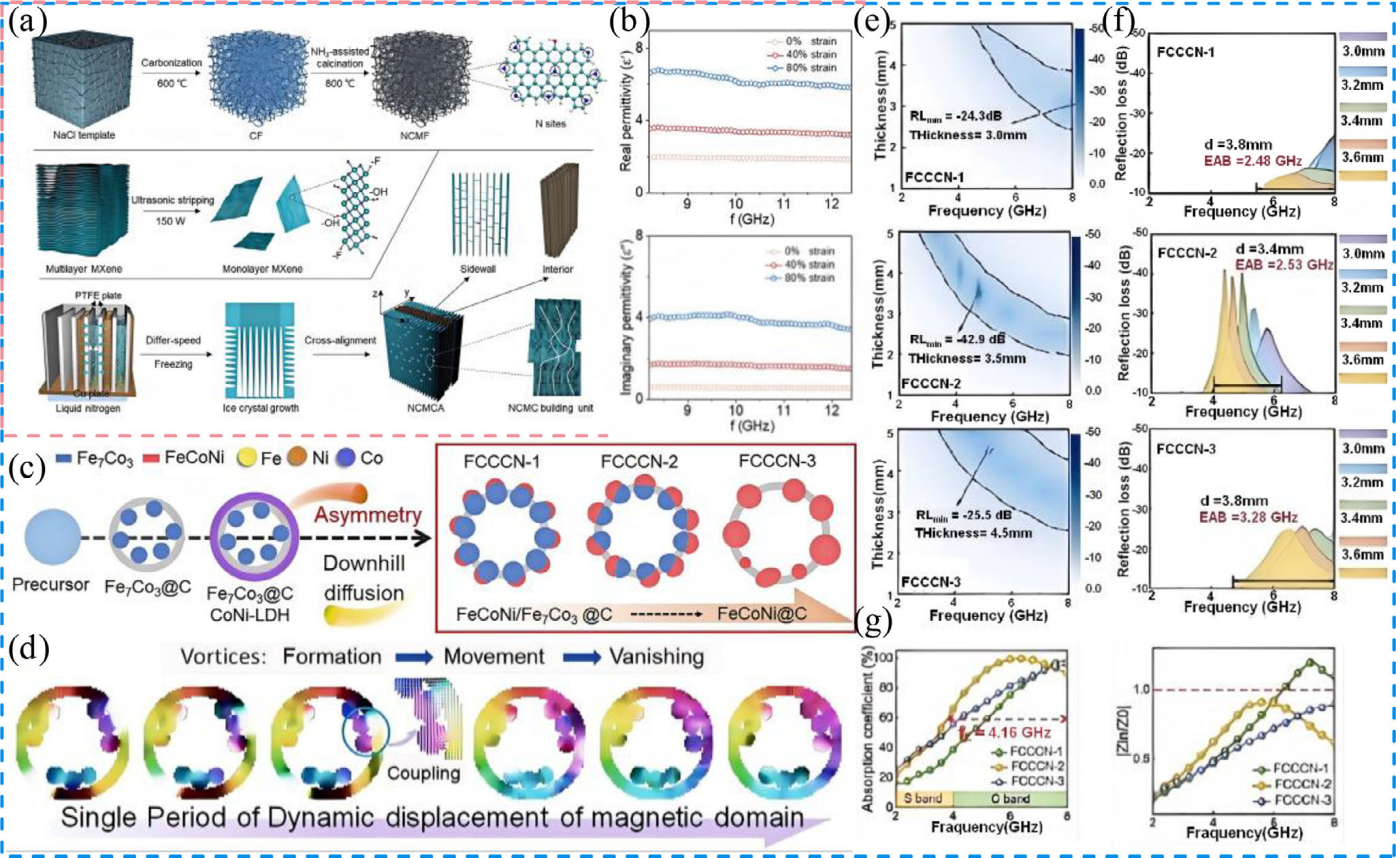
(a) The schematic preparation of the sandwich NCMCA, (b) change diagram of *ε*' and *ε'*' [48] Copyright 2025, Wiley, (c) asymmetric atom diffusion to fabricate FeCoNi/Fe_7_Co_3_ nano‐interface heterostructures, (d) distribution of the magnetic moments of the core–shell microtubes under a 6.0 GHz electromagnetic field acquired from the Mumax3 simulation, (e) 2D color mapping of R_L_ values of FCCCN‐x samples, (f) Effective absorption bandwidth at different thicknesses of FCCCN‐x samples, (g) comparison of absorption coefficients and impedance matching between FCCCN‐x samples with a thickness of 2.8 mm [49] Copyright 2025, Wiley.

The synergistic modulation of equivalent permittivity (*ε*_r_) and the permeability (*µ*_r_) in porous structures is achieved through the spatial reconstruction of gas‐solid interfaces (Figure 5a–d) [48, 49]. Parameters such as porosity, pore morphology, and particle distribution critically influence impedance matching and absorption peak positions by tuning the real and imaginary components of electromagnetic parameters (Figure 5e–g). Introducing porosity gradients or multilevel pores along the wave propagation direction triggers phase perturbations in interfacial reflections, reducing overall reflectivity via coherent phase cancellation. Simultaneously, surface sub‐wavelength pores disrupt specular reflection, promoting scattering into the material interior. At the microscopic level, intricate pore walls induce multiple scattering and refraction, extending the effective propagation path and enhancing energy attenuation per unit thickness. Furthermore, gas‐solid heterogeneous interfaces within pores generate additional relaxation and magnetic losses through interfacial polarization, charge accumulation, and magnetic domain distortion.

Porous structures, often arising from lattice growth instability or defect aggregation, feature unsaturated bonds at pore edges that act as polarization centers, inducing local electron valence changes and potential differences that contribute to interface loss. The increased specific surface area exposes more atoms as active sites for interfacial reactions, initiating electromagnetic energy conversion. Theoretical modeling suggests that while increased porosity generally lowers the real part of the equivalent dielectric constant, the local field enhancement effect at pore‐wall interfaces can simultaneously boost both the real (*ε*') and imaginary parts of permittivity and permeability. This combination establishes a “low‐reflection, high‐loss” synergistic mechanism. In multi‐layer designs, integrating the dispersion characteristics of dielectric parameters with surface phase modulation and intermediate gradient impedance transitions is essential for broadband matching. The electromagnetic behavior of these porous systems is described by effective medium theory and frequency‐domain magnetic loss models, representing a spatial superposition of polarization, resonance, and eddy‐current loss.

**FIGURE 6:**
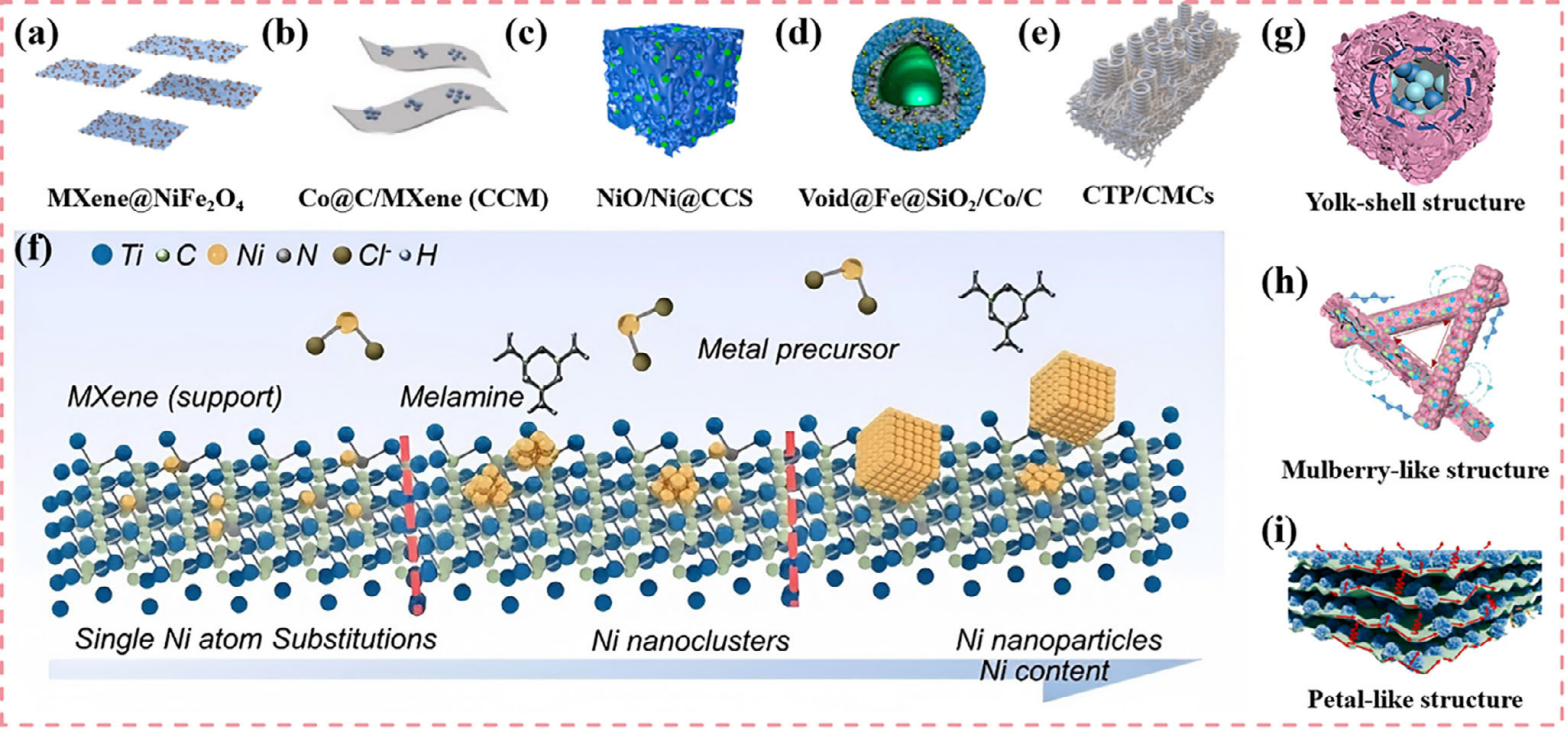
(a) Preparation of MXene@NiFe_2_O_4_ [51], Copyright 2025, Springer, (b) synthesis of Co@C/MXene (CCM) [52], Copyright 2024, Elsevier B.V., (c) production of NiO/Ni@CCS [53], Copyright 2025, Elsevier B.V., (d) structure of void@Fe@SiO_2_/Co/C [62], Copyright 2024, Elsevier B.V., (e) structure of CTP/CMCs [63], Copyright 2025, Elsevier B.V., (f) schematic representation of the fabrication process for Ni‐MX [64], Copyright 2025, Springer, (g) yolk–shell structure [65], Copyright 2025, Elsevier B.V., (h) Mulberry‐like 1D core–shell structured Fe@Ni_(x)_ nanowires (NWs) [54] Copyright 2024, Elsevier B.V., (i) flower‐like CoNi nanosheets/nitrogen‐doped expanded graphite (CoNi/N‐EG) composites [56] Copyright 2025, Elsevier B.V.

However, it is imperative to acknowledge that significant challenges persist for porous wave‐absorbing materials regarding pore controllability, structural homogeneity, and the discrepancy between theoretical models and experimental outcomes. The study of the structure and material itself is not limited to its simple physical composite, incorporating mono, binary [50], or even multivariate forms of Fe [51], Co [52], and Ni [53]. It also includes the optimization of structures such as mulberry‐like [54], petal‐like [55, 56], and spiderweb‐like [57] (Figure 6). These architectures undergo specific chemical transformations during synthesis, creating inhomogeneous electron cloud distributions under electric fields that enhance loss performance. However, inherent properties of metal particles yield divergent outcomes; conventional metal/ferrite‐based porous materials often suffer from high density and poor low‐frequency absorption, hindering the balance between lightweight and broadband requirements. While 3D conductive networks enhance dielectric loss, they frequently cause an imbalance between dielectric and magnetic losses. Moreover, ultra‐high temperature carbonization or calcination can compromise structural stability, leading to skeletal collapse and degraded electromagnetic performance. Consequently, few materials simultaneously satisfy the criteria of being thin, lightweight, broadband, and strong, as optimizing one property often compromises others.

To overcome these limitations, research has increasingly focused on carbon‐based porous systems, particularly carbon nanofibers (CNFs) [58]. Owing to their 1D structure, high aspect ratio, controllable pore size distribution, and abundant surface functional groups, CNFs enable continuous conductivity and precise pore structure control [59]. Their advanced dielectric‐magnetic synergistic capabilities and substantial specific surface area effectively enhance broadband absorption strength and impedance matching [60, 61]. Consequently, the advent of carbon nanofibers has spurred a new trend in the development of high‐performance electromagnetic wave‐absorbing materials.

## 3 1D Carbon Nanofibers: Conductive Networks and Anisotropy

While traditional reviews often categorize carbon materials broadly, this section specifically isolates the unique functionality of 1D carbon nanofibers (CNFs) [66, 67]. Typically synthesized via electrospinning followed by stabilization and carbonization, 1D CNFs stand in distinct contrast to their 0D (carbon black [68]) or 2D (graphene [69, 70]) counterparts. Their high aspect ratio and unique topology enable the construction of long‐range conductive networks with minimal percolation thresholds. This geometric advantage is pivotal for developing lightweight EWAMs, as it allows for efficient conductivity at ultra‐low filler loadings‐addressing the critical demand for density reduction in aerospace and portable electronic applications.

However, the electromagnetic performance of pristine CNFs is frequently constrained by their inherent high conductivity. This often results in severe impedance mismatch, causing dominant wave reflection rather than the desired absorption. Consequently, this section critically analyzes strategies employed to tailor the microstructure and composition of CNFs. The goal is to strike an optimal balance between conduction loss and polarization mechanisms, thereby transforming CNFs from highly reflective conductors into efficient absorbers.

### 3.1 Advanced Architectural Designs: From Heteroatom Doping to Hierarchical Composites

To address the intrinsic impedance mismatch challenges of pristine carbon nanofibers (CNFs), recent research has pivoted from simple material selection to precise architectural engineering. Strategies such as heteroatom doping, defect engineering, and the construction of multiscale hybrid interfaces have transformed CNFs from passive conductive fillers into active “conductive backbones” capable of synergistic loss mechanisms [71, 72, 73, 74, 75, 76, 77]. A primary approach involves optimizing the conductive network topology to prevent localized charge aggregation while maintaining efficient electron transport. For instance, introducing carbon nanotubes (CNTs) alongside CNFs creates a unique “bridging effect,” where CNTs span gaps between entangled fibers to enhance conductivity without compromising structural integrity [78, 79]. Beyond pure carbon architectures, integrating magnetic metal nanoparticles (e.g., Fe, Co, Ni) introduces a multi‐mechanism loss system. These inclusions provide magnetic loss via natural resonance while generating significant interfacial polarization at the heterogeneous boundaries between metal cores and carbon sheaths [80, 81, 82].

**FIGURE 7:**
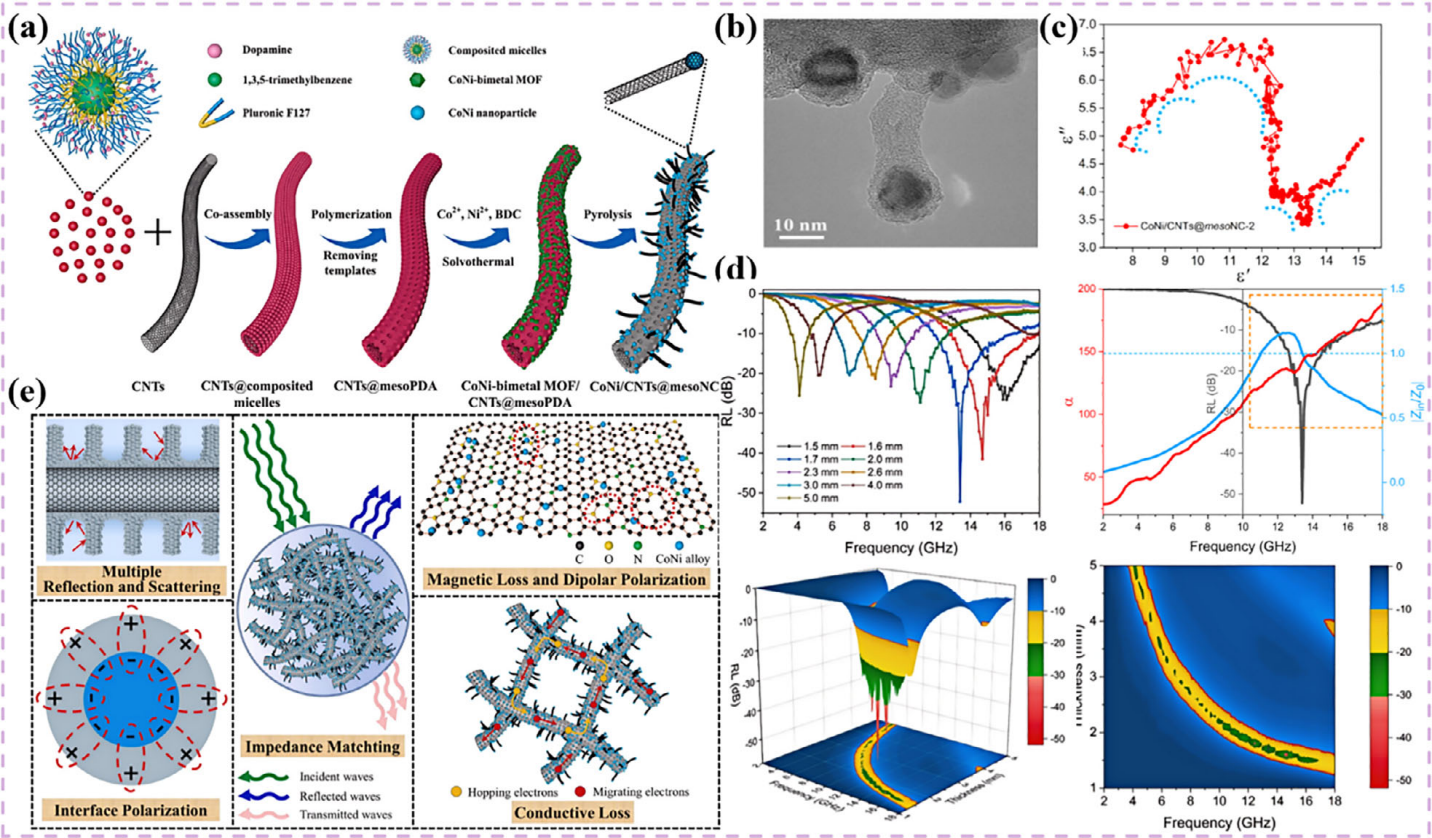
(a) Preparation of CoNi/CNTs@mesoNC nanofibers, (b) Microwave absorption mechanisms of CoNi/CNTs@mesoNC composites, (c) Scanning electron microscope images of CoNi/CNTs@mesoNC, (d) Cole‐Cole plots of the CoNi/CNTs@mesoNC nanofibers, (e) *R*_L_ curves, 3D *R*_L_ plots, and corresponding 2D contour *R*_L_ maps and the frequency‐dependent |Z_in_/Z_0_|, α, and R_L_ values at the 1.7 mm thickness of CoNi/CNTs@mesoNC‐2 [84], Copyright 2023, Elsevier B.V.

Representative studies highlight the power of this hierarchical design. Zhang et al. [83] fabricated core–sheath structured Co‐Fe@C/SiO_2_ CNFs, where uniformly embedded magnetic nanoparticles acted as loss centers, and the SiO_2_ sheath facilitated impedance gradient matching. This architecture achieved a minimum reflection loss (RL) of ‐59.6 dB with a bandwidth of 4.6 GHz at an ultra‐thin thickness of 1.43 mm. Similarly, Wei et al. [84] developed a complex CoNi/CNTs@mesoNC composite featuring a mesoporous carbon shell and nitrogen‐doped CNTs (Figure 7). By integrating mesoporous engineering, heterogeneous atom doping, and magnetic modulation, this design created abundant polarization sites and optimized conduction paths, yielding an RL of ‐52.1 dB and a broadband absorption of 5.2 GHz. These examples demonstrate that dimensional synergy and interface engineering are key to overcoming the limitations of single‐component CNFs.

In wave‐absorbing materials, the ordered 3D spatial arrangement of CNFs constructs multiscale pores and channels, inducing a large number of electrons in polarization centers of heterogeneous interfaces along the optimal path of the leaping behavior. The inter‐fiber gap can be formed by the gradient of heterogeneous interfaces and the realization of the multiscale electromagnetic scattering, which in turn affects the various loss indicators. The synergistic design of anisotropy and multilevel structure is expected to overcome the inherent contradiction between high dielectric loss and low impedance‐matching in traditional disordered packing systems. Furthermore, it offers a new approach to developing thin, lightweight, wide, and strong materials.

### 3.2 Structural Optimization of CNFs: Confinement Strategies and Dimensional Hybridization

As high‐performance wave‐absorbing materials, carbon nanofibers (CNFs) offer a structural basis for tuning electromagnetic polarization through their orientable, flexible, and tunable 1D networks. These advantages make CNFs ideal candidates for lightweight, broadband aerospace coatings. However, the practical application of pure CNFs faces significant bottlenecks. The high aspect ratio of CNFs, while beneficial for conductivity, often leads to localized entanglement during electrospinning, destroying the uniformity of the 3D conductive network. Furthermore, the limited surface functional groups on CNFs restrict chemical bonding, causing interfacial stripping that attenuates both mechanical properties and dielectric loss. Additionally, excessive orientation enhances electron conduction but reduces multi‐level scattering, limiting overall attenuation capability. To overcome these inherent limitations, CNF‐based strategies have evolved towards microscopic confinement and macroscopic dimensional hybridization.

#### 3.2.1 Microscopic Confinement: Optimizing Internal Magnetic Distribution in CNFs

**FIGURE 8:**
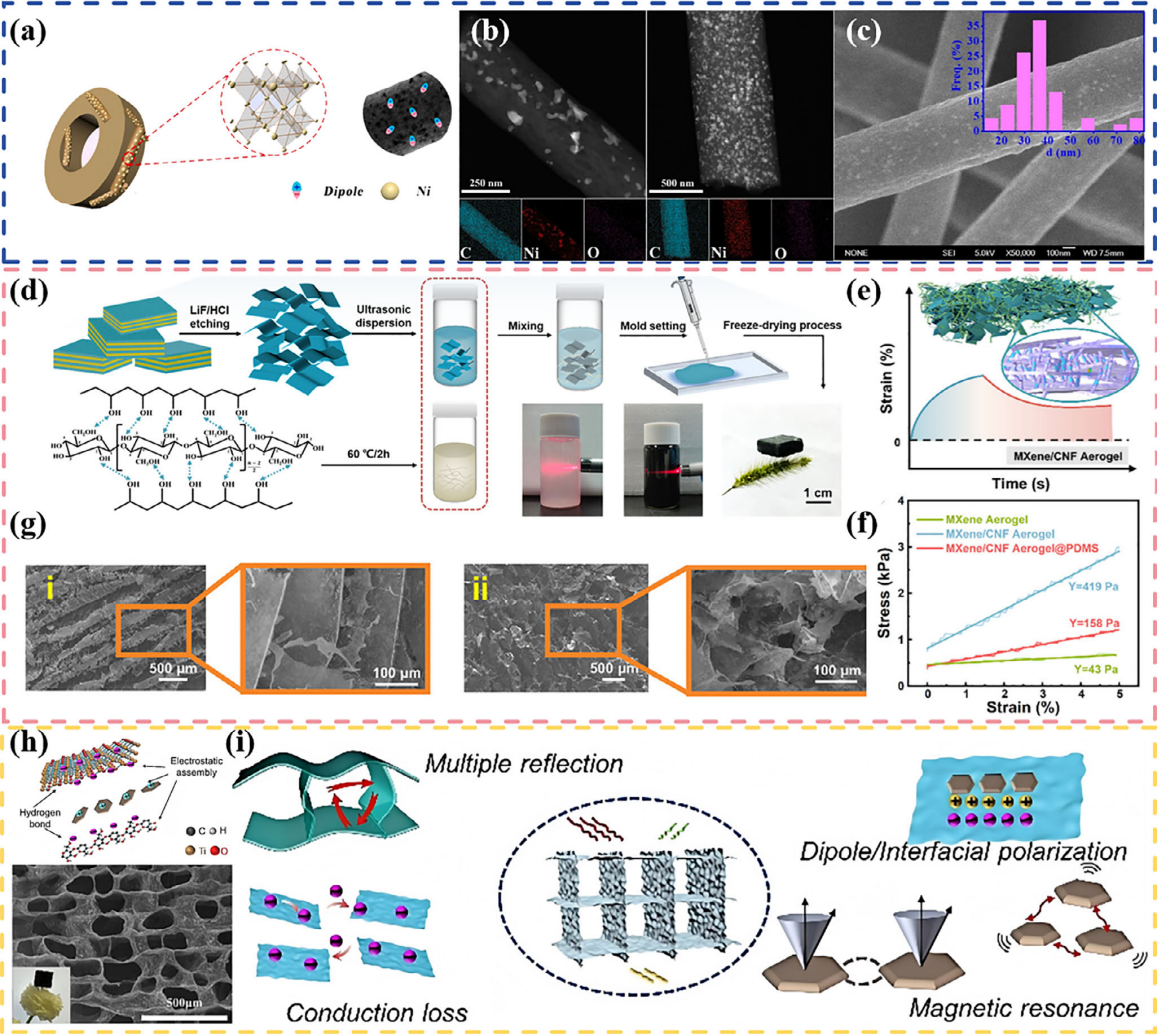
(a) MOF space restriction strategy, (b) uniform distribution of C, O elements and NiNPs in carbon fibers, (c) grain size distribution map of S3 [85], Copyright 2025, Elsevier B.V., (d) preparation of MXene/CNF aerogel, (e) MXene/CNF aerogel, (f) Young's modulus of MXene aerogel, MXene/CNF aerogel and MXene/CNF aerogel@PDMS, (g) highly entangled and overlapping 3D structure formed by the combination of MXene and CNF [90], Copyright 2025, Wiley, (h) electrostatic interaction and hydrogen bonding effect between MXene, Fe_3_O_4_ nanodisks and CNF and honeycomb porous structure, (i) Schematic diagram of electromagnetic wave absorption mechanism of CMFA composite material [89], Copyright 2023, Elsevier B.V.

To address the issue of magnetic nanoparticle agglomeration within the CNF matrix‐a common defect that degrades impedance matching‐researchers have employed spatial confinement strategies. Shen et al. [85] utilized a MOF spatial confinement strategy [86, 87] specifically to regulate the growth of magnetic components inside the CNFs. By preventing the agglomeration of Ni nanoparticles during precursor formation, this approach ensured the uniform distribution of magnetic nickel nanoparticles within the CNF backbone (Figure 8a,b). Consequently, the CNF‐based composite achieved an excellent absorption bandwidth of 5.22 GHz in the Ku‐band at an ultra‐low filler loading of 3 wt%. The significantly reduced coating density (Figure 8c) demonstrates how optimizing the internal microstructure of CNFs can effectively counteract radar detection risks while maintaining the lightweight design essential for aerospace applications

#### 3.2.2 Macroscopic Hybridization: Reinforcing CNF Networks With 2D Materials

While microscopic optimization improves internal homogeneity, the structural instability and weak interfacial bonding of CNF networks require macroscopic reinforcement. Although carbon nanotubes (CNTs) share similar conductive properties, their tendency to form inhomogeneous networks and lack of sufficient surface functionality limits their ability to solve the multiscale scattering problems of CNFs. Therefore, CNFs have been increasingly hybridized with MXenes, whose rich surface groups and large specific surface area serve to complement the deficiencies of the CNF architecture [88].

In this composite strategy, MXenes act not as replacements but as functional supporters for the CNF network. Specifically, this synergy manifests in two key aspects: 1. Structural Stabilization: The oriented arrangement of CNFs can lead to network collapse; however, introducing MXene nanosheets creates a “line‐to‐surface” support structure. In this configuration, CNFs provide strong longitudinal scaffolding that prevents MXene restacking while enhancing the composite's resilience and Young's modulus (Figure 8d–f). 2. Interface Enhancement: The scarce functional groups on pure CNFs often lead to interfacial failure. The abundant surface terminations modify the local electronic structure and promote interfacial charge transfer, thereby enhancing dielectric polarization and energy dissipation (Figure 8g).

The synergistic potential of this CNF‐reinforced architecture was exemplified by Wang et al. [89], who assembled a 3D porous aerogel using a CNF backbone combined with Ti_3_C_2_T_x_ MXene and Fe_3_O_4_ nanodiscs. In this system, the CNFs inhibited the interlayer agglomeration of MXene and strengthened interfacial bonding through hydrogen/electrostatic interactions. The resulting composite leveraged the abundant conduction channels and active reaction sites at the CNF/MXene interface, combined with the magnetic loss of Fe_3_O_4_, to optimize multiple loss mechanisms (Figure 8h,i). This CNF‐based hierarchical structure achieved a minimum reflection loss of ‐56.8 dB and a broadband absorption of 6.68 GHz under ultra‐thin conditions. These advancements confirm that by strategically integrating auxiliary components like MOFs and MXenes, the inherent limitations of CNFs can be transformed into superior wave‐absorbing performance, establishing a firm foundation for their application in sensors, shielding, and stealth technologies.

## 4 2D Layered Polarization & Surface Termination Engineering

Transitioning from the 1D conductive networks of CNFs, this section explores the distinct absorption mechanisms of 2D MXenes, where the focus shifts towards interfacial polarization within layered structures and dipole polarization induced by surface terminations. We highlight how the atomic‐scale thickness of MXenes offers unique advantages in lightweight applications but simultaneously poses significant challenges regarding stacking‐induced performance degradation and oxidative instability. This discussion is critical for understanding how the planar confinement of 2D materials complements the linear pathways of 1D fibers. Specifically, it sets the stage for later discussions on how combining 2D MXenes with 1D CNFs can synergistically optimize both attenuation capabilities and impedance matching, overcoming the inherent limitations of single‐dimensional architectures.

### 4.1 MXene‐Based Absorbers With Interface and Structural Engineering

**FIGURE 9:**
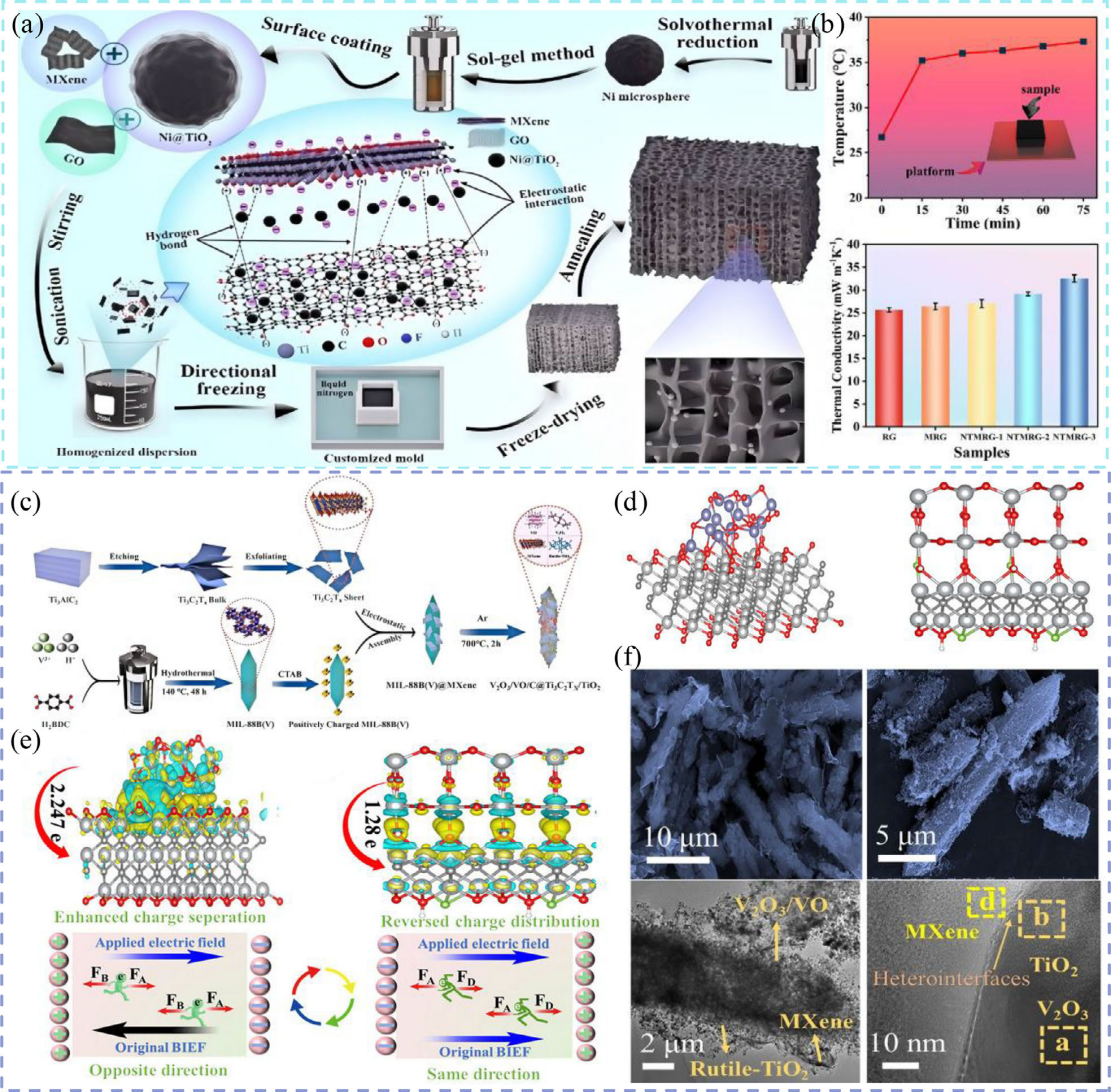
(a) Schematic diagram of the preparation of NTMRG aerogels, (b) *T*–*t* curves of NTMRG aerogels and thermal conductivity of RG, MRG, NTMRG‐1, NTMRG‐2 and NTMRG‐3 aerogels [99] Copyright 2024, Elsevier B.V., (c) schematic diagram of the preparation process of V_2_O_3_/VO/C@Ti_3_C_2_T_x_/TiO_2_ composites, (d) the model of V_2_O_3_/VO/MXene and TiO_2_/MXene, (e) analysis of the difference of the charge density and charge transport mechanism analysis, (f) SEM images of MIL‐88B(V)@MXene and SEM, TEM and HRTEM images of MM‐700 [88] Copyright 2025, Wiley.

MXenes, a family of 2D transition metal carbides, nitrides, and carbonitrides with the general formula M*_n_*_+1_X*_n_* T*_x_*, (where M is a transition metal like Ti, Mo, or V; X is C or N, and T_x_ represents surface terminations such as ─O, ─OH, or ─F) [91, 92], are considered highly promising candidates for electromagnetic wave absorption due to their alternating atomic layer structure and tunable dielectric properties. Their excellent performance stems from atomic‐thin layers (≈1 nm) that provide high specific surface area and rich interlayer interfaces for multiple reflections, alongside a strong covalent bonding network of transition metals and carbon/nitrogen atoms that facilitates conduction loss via off‐domain electrons [93, 94, 95, 96], carbon, and nitrogen atoms form a strong covalent bonding network. Free carriers within the layers generate conduction currents when an electromagnetic field is applied, causing energy dissipation. Conversely, the abundant functional groups (─O, ─OH, and ─F) and structural defects on the MXene surface can act as polarization centers, inducing dipole polarization. The relaxation process increases dielectric loss, which improves the material's wave‐absorbing properties [97]. While microscopic regulation optimizes intrinsic loss mechanisms, the macroscopic assembly of MXenes into hierarchical architectures is essential to prevent restacking and introduce additional loss dimensions, such as magnetic loss and porous scattering. Pure MXene films often suffer from dense packing, which limits electromagnetic wave penetration and exacerbates the skin effect. Breaking through these bottlenecks requires a multiscale, synergistic design approach that introduces magnetic and dielectric materials to construct heterogeneous interfaces [49, 98]. Creating macroporous structures is a dominant strategy to extend the propagation path of electromagnetic waves through multiple internal reflections. Shao et al. [99] exemplified this by constructing a hierarchical porous structure through embedding Ni@TiO_2_ core–shell microspheres into an rGO/MXene backbone using a directional freeze‐drying technique (Figure 9a). In this multiscale design, the Ni core provides magnetic loss, the TiO_2_ shell layer enhances dielectric loss through interfacial polarization, and the MXene nanosheets form a hydrogen‐bonding network with rGO through surface functional groups (─OH, ─F), effectively preventing graphene stacking and optimizing conductive pathways. This structural design enhances the reflection and polarization loss of electromagnetic waves, achieving good impedance matching through component modulation. The resulting Ti@TiO_2_/MXene/rGO aerogel demonstrated excellent wave‐absorbing performance, with an EAB of 6.48 GHz (39% broader than pure rGO aerogel at 4.64 GHz) and a deep *R*_L_ of ‐71.4 dB, reaching a level higher than that of similar MXene‐based materials (Figure 9b). This work highlights how macroscopic architectural design can effectively mitigate the stacking issue inherent to 2D materials.

### 4.2 Macroscopic Assembly: Hierarchical Architectures and Hybrid Composites

Beyond macroscopic porosity, the construction of multidimensional heterostructures at the microscopic level can further increase the number of refractions and reflections of incident electromagnetic waves within a material, multiplying the loss paths. This approach leverages the built‐in electric field at heterogeneous interfaces to induce a redistribution of carriers, accelerating carrier jumps and enhancing dielectric polarization. Zhai et al. [88] advanced this strategy by preparing V_2_O_3_/VO/C@Ti_3_C_2_T_x_/TiO_2_ multidimensional hetero‐structured composites via electrostatic self‐assembly, compositing modified MIL‐88B(V) with MXene in a temperature‐modulated carbonization process (Figure 9c,d). By modulating the polarity difference of surface groups, the complex permittivity

(6)

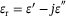




(Equation 6) is tuned to optimize impedance matching. The multidimensional interfaces formed by MXene with V_2_O_3_/VO and TiO_2_ spontaneously generate a built‐in electric field through the equilibrium of Fermi energy levels, inducing strong interfacial polarization loss (Figure 9e).

**FIGURE 10:**
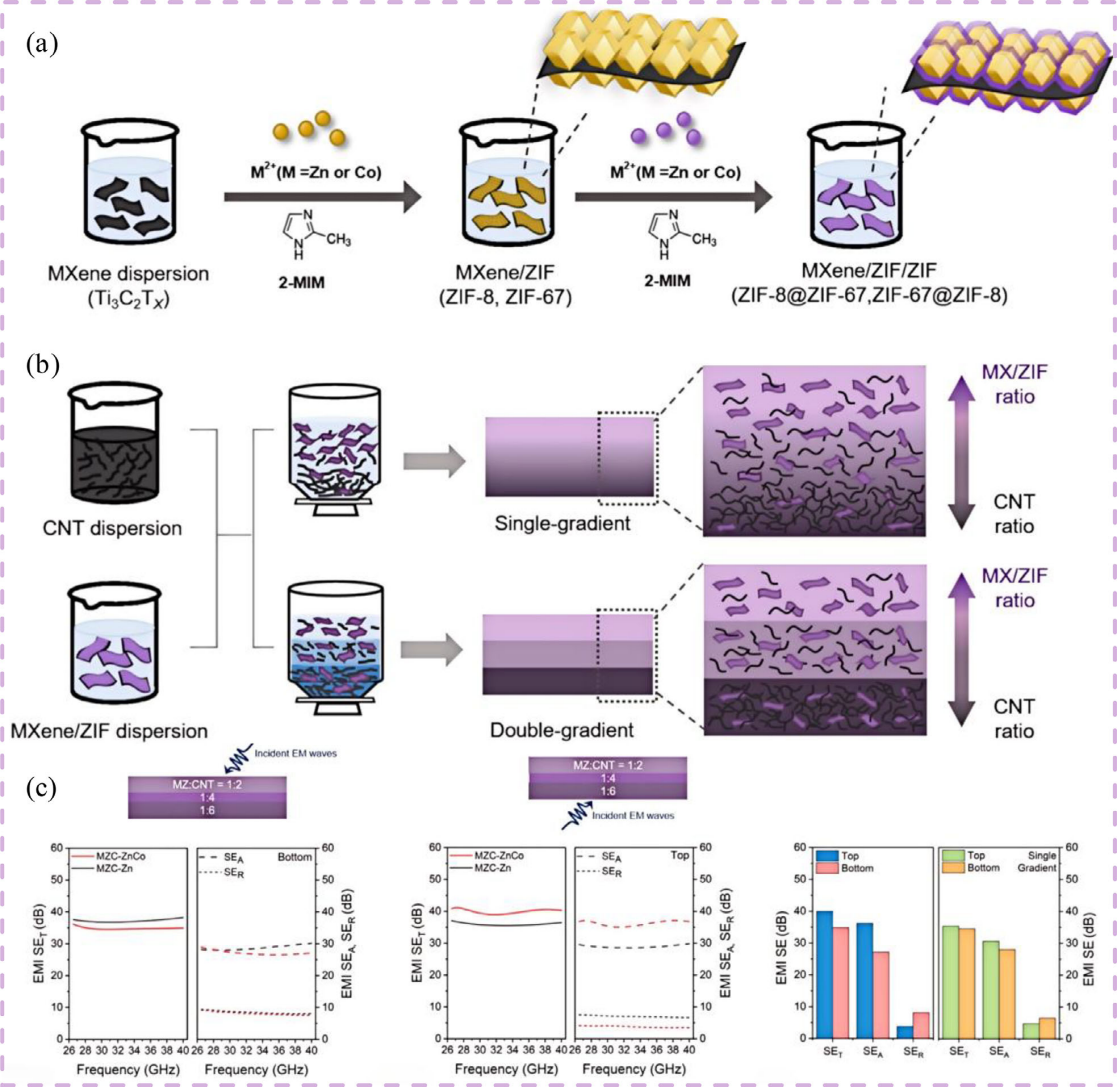
(a) Schematic illustration for the synthesis of MXene/ZIF hybrid nanosheets in a solvent, (b) schematic illustration for the fabrication of gradient‐structured MXene/MOF/CNT films for absorption‐enhanced EMI shielding, (c) total shielding effectiveness (SET), reflection (SER), and absorption (SEA) components of dual gradient films at Ka‐band [100] Copyright 2025, Elsevier B.V.

Simultaneously, the multidimensional network formed by the MOF‐derived carbon skeleton and MXene optimizes the carrier‐leaping path (Figure 9f). This microscopic defect and interface engineering enabled the composite to achieve an excellent EAB of 6.16 GHz and a minimum *R*_L_ of ‐50.10 dB at a 20% filling ratio. Furthermore, integrating MXenes into gradient structures allows for simultaneous shielding and absorption in flexible electronic applications. Jang et al. [94] fabricated MXene/ZIF‐8/ZIF‐67/CNT composite films with a vertical gradient distribution, where the top layer rich in porous MOFs facilitated wave entry and absorption through scattering, while the bottom CNT‐rich layer provided a conductive network for shielding (Figure 10a,b). This dual‐function film achieved a total shielding efficiency of 40 dB with a low reflection rate, offering a viable solution for low‐interference EMI shielding in high‐frequency communication devices (Figure 10c).

Despite significant progress via surface engineering and hybrid composites, MXenes face dual bottlenecks: intrinsic instability and structural limitations. Prone to oxidation in humid environments, MXenes suffer from degraded functional groups and collapsed interlayers, which weaken loss channels and narrow absorption bandwidths [101]. Furthermore, their reliance on hazardous, high‐cost HF‐based etching hinders scalable, green production [102], necessitating urgent developments in gentle synthesis and protective coating strategies [103, 104]. Compounding these chemical challenges is the physical issue of nanosheet restacking, which reduces active sites and limits spatial configuration, often requiring complex external templates. To simultaneously resolve these issues‐enhancing stability, enabling scalable fabrication, and maximizing internal voids for multiple scattering‐a shift toward intrinsic 3D porous architectures is imperative. This naturally leads to MOF‐derived 3D carbonaceous frameworks, whose pre‐designed topologies and abundant active sites offer an ideal robust scaffold to host MXenes (preventing restacking and oxidation) or function as standalone high‐performance absorbers, paving the way for next‐generation electromagnetic materials.

## 5 Porous MOF‐Derived Hybrids for Tunable Microwave Absorption

Finally, we examine 3D MOF‐derived architectures, which introduce a critical third dimension of complexity: multiple scattering and hierarchical porosity. Unlike the linear conductive pathways of 1D fibers or the planar confinement of 2D MXenes, 3D porous frameworks fundamentally extend the propagation path of incident electromagnetic waves, enhancing energy dissipation through repeated internal reflections and refractions. This section evaluates how the tunable pore size, topology, and composition of MOF derivatives address the specific limitations identified in previous sections‐namely, the structural simplicity of CNFs and the restacking‐induced instability of MXenes. By leveraging intrinsic voids and abundant active sites, MOF‐derived hybrids serve not merely as absorbers but as versatile 3D scaffolds that optimize impedance matching while maximizing attenuation capabilities, paving the way for next‐generation lightweight and broadband materials. Following the development of carbon‐based and MXene systems, metal‐organic frameworks (MOFs) have emerged as a distinct class of crystalline porous materials composed of metal ions or clusters self‐assembled with organic ligands via coordination bonds. Their regularly arranged nodes, diverse topologies, and tunable structural units place them at the forefront of wave‐absorbing material design. The following analysis details how structural modulation and component engineering in MOF‐derived hybrids overcome the bottlenecks of single‐component absorbers.

### 5.1 Structural Advantages and Microscopic Regulation

**FIGURE 11:**
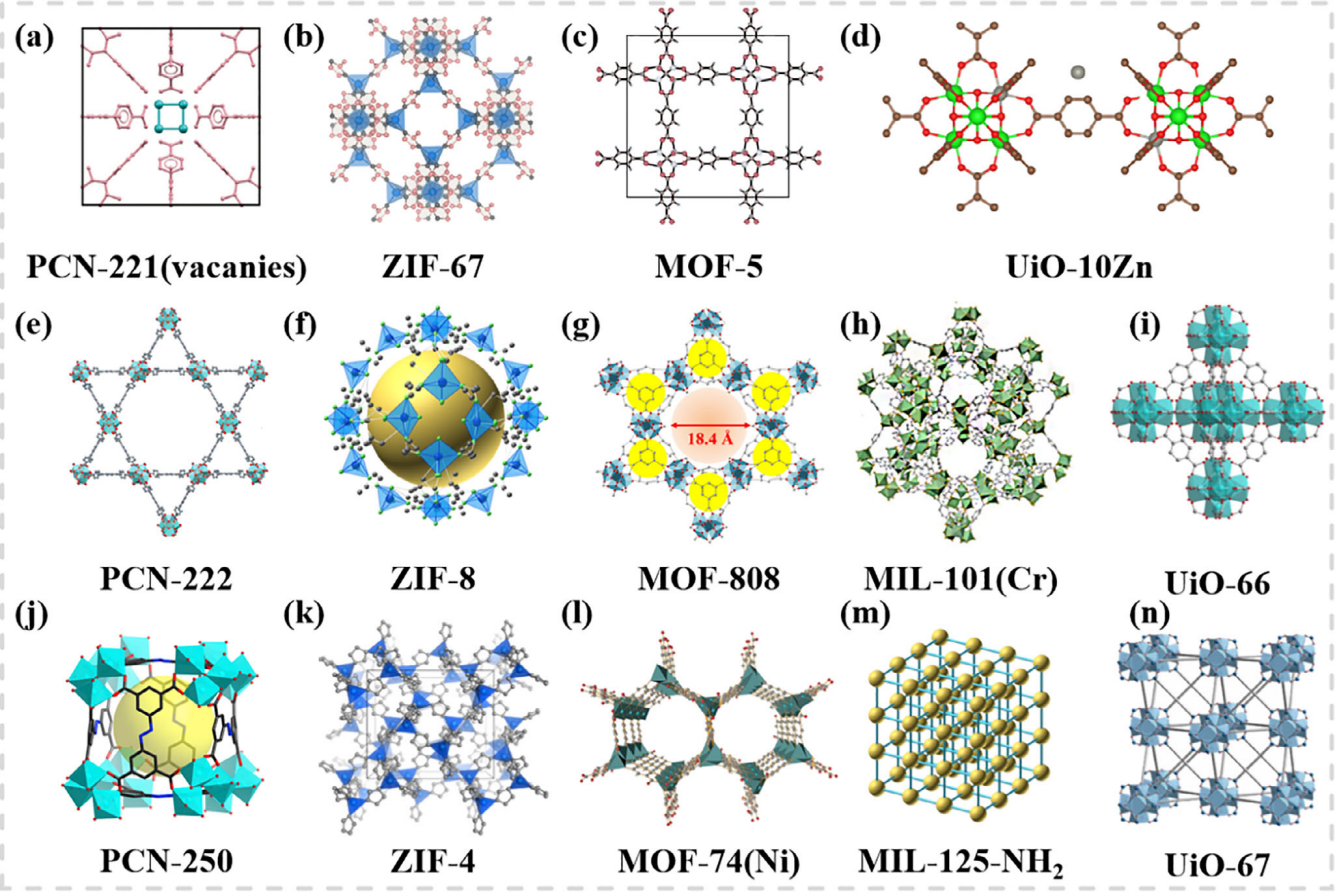
Schematic diagram of the structure of various MOFs (a) PCN‐221 [112], Copyright 2021, Springer, (b) ZIF‐67 [113], Copyright 2021, Elsevier B.V., (c) MOF‐5 [110], Copyright 2022, Elsevier B.V., (d) UIO‐10(Zn) [114] Copyright 2025, Elsevier B.V., (e) PCN‐222 [115], Copyright 2024, American Chemical Society., (f) ZIF‐8, (g) MOF‐808 [116], Copyright 2025, Elsevier B.V., (h) MIL‐101(Cr) [117] Copyright 2025, Wiley., (i) UiO‐66 [118] Copyright 2025, Elsevier B.V., (j) PCN‐250 [107], Copyright 2017, Elsevier B.V., (k) ZIF‐4 [119] American Chemical Society., (l) MOF‐74(Ni) [120], Copyright 2024, Wiley., (m) MIL‐125‐NH_2_, (n) UIO‐67 [121] Copyright 2025, Elsevier B.V.

The exceptional performance of MOF‐derived materials stems from their multiscale porous architecture (micro‐, meso‐, and macropores), which facilitates wave penetration and multiple internal reflections [105]. Depending on the types of metal centers and organic ligands, MOFs can be derived from several series of materials, such as ZIF [106], UIO, PCN [107], CPL [108], MIL [109], and IRMOF [110] (Figure 11). The ZIF and MIL series are especially common in wave absorption research due to their excellent properties [111].

**FIGURE 12:**
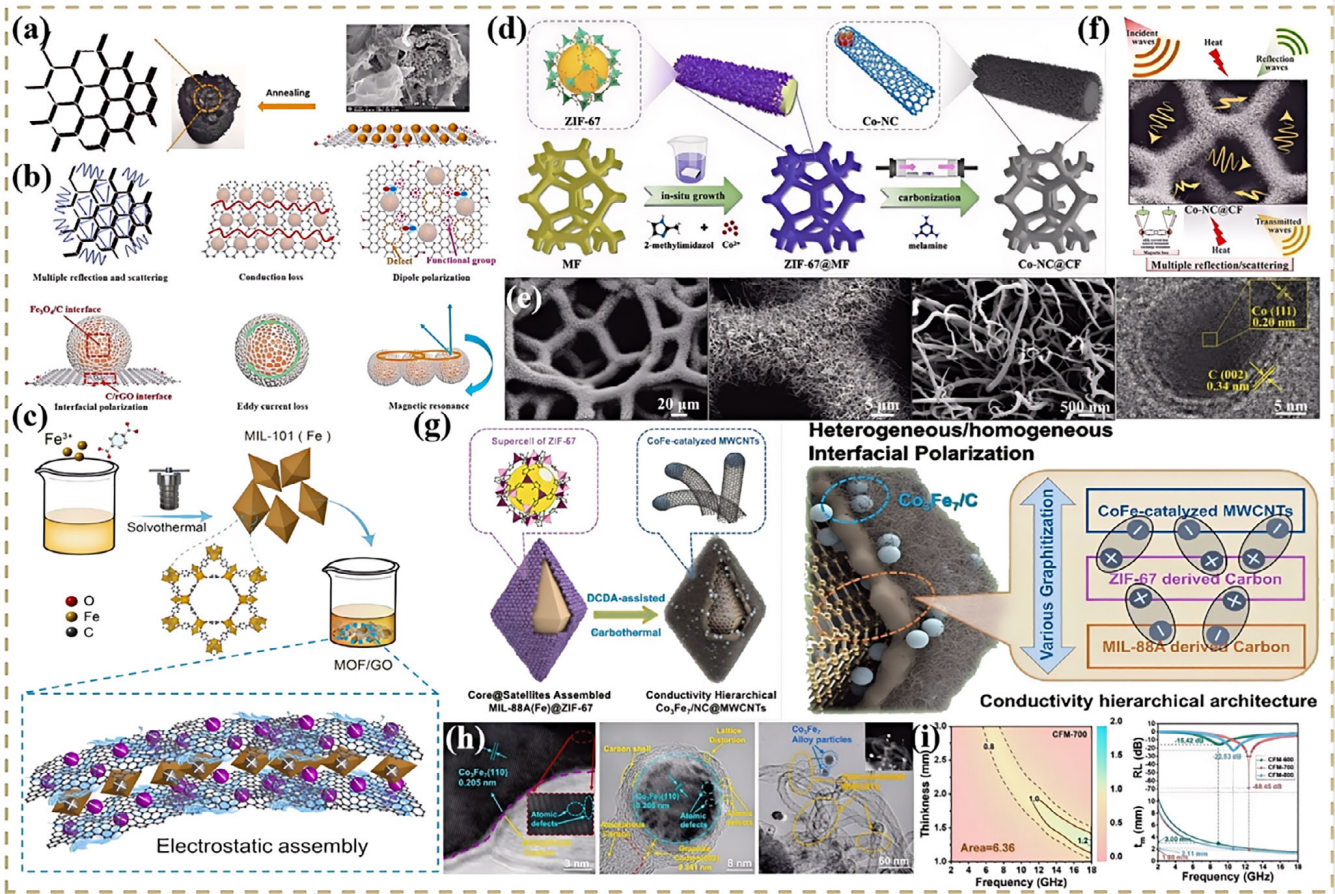
(a) Schematic diagram of the structure of Fe‐rGO composite aerogel before and after annealing, (b) multiple loss mechanisms of materials under the influence of single‐crystal magnetic Fe_3_O_4_ nanospheres [122] Copyright 2024, Elsevier B.V., (c) a special regular structure integrating MOF‐derived core–shell γ‐Fe_2_O_3_@C particles with graphene layers [123] Copyright 2023, Springer, (d) Schematic diagram of the preparation process of Co‐NC@CF, (e) SEM images of Co‐NC@CF‐800, (f) Loss benefits brought about by structural changes and the ordered spin process of the π electron cloud [106] Copyright 2025, Springer, (g) magnetic‐dielectric synergistic effect achieved by heterogeneous interface and dual ligand thermal decomposition, (h) HRTEM images of CFM‐700 sample, (i) three CFM sample *R*_L_ values and best sample individual *R*_L_ presentation [125] Copyright 2025, Elsevier B.V.

Unlike conventional materials, MOF pyrolysis generates in‐situ metal/metal oxide nanoparticles embedded within a porous carbon matrix, creating abundant heterogeneous interfaces that synergize dielectric and magnetic losses. Controlling crystallinity and macroscopic assembly is crucial for minimizing defect interference. For instance, Cao et al. [122]. synthesized single‐crystalline magnetic Fe_3_O_4_ nanorods integrated into rGO aerogels (600Fe‐rGO) via temperature‐controlled calcination. The single‐crystal structure reduced grain‐boundary scattering, while d‐p orbital hybridization altered localized electron distribution to enhance interfacial polarization, achieving excellent absorption at a low filling ratio of 5 wt% (Figure 12a,b). Similarly, Lin et al. [123]. developed an ultra‐lightweight, 3D, porous aerogel using electrostatic assembly and thermal annealing. This aerogel integrates MOF‐derived, core–shell, γ‐Fe_2_O_3_@C particles with graphene layers. It can achieve a reflection loss of up to 60.5 dB and an EAB of up to 7.76 GHz through synergistic conduction loss, interfacial polarization, and magnetic dielectric coupling (Figure 12c).

Additionally, defects, charge buildup, and uneven charge distribution often form localized electric fields at interfaces, inducing incident electromagnetic waves and further energy conversion. Additional benefits can be realized for electromagnetic losses through structural designs such as porous and helical layered designs [124]. Guo et al. [106]. designed multi‐layer heterostructures (Co/CNT, CNT/CF, Co‐N‐C) to create electric field gradients (Figure 12d), which induced strong interfacial polarization and enhanced dielectric loss, while the ordered arrangement of π‐electrons enhanced conductive loss and interfacial polarization (Figure 12e,f). Furthermore, coupling different MOFs addresses the issue of single loss mechanisms, Cai et al. [125]. combined Fe‐MOF and Co‐MOF to achieve a magnetic‐dielectric synergistic effect. This was done by forming a multilevel conductive structure through heterogeneous interfaces and dual ligand pyrolysis (Figure 12g,h). The result was an ultra‐low reflection loss over a wide frequency band and a broad adequate absorption bandwidth (Figure 12i).

### 5.2 Component Modulation: From Bimetallic to Ternary Alloys

**FIGURE 13:**
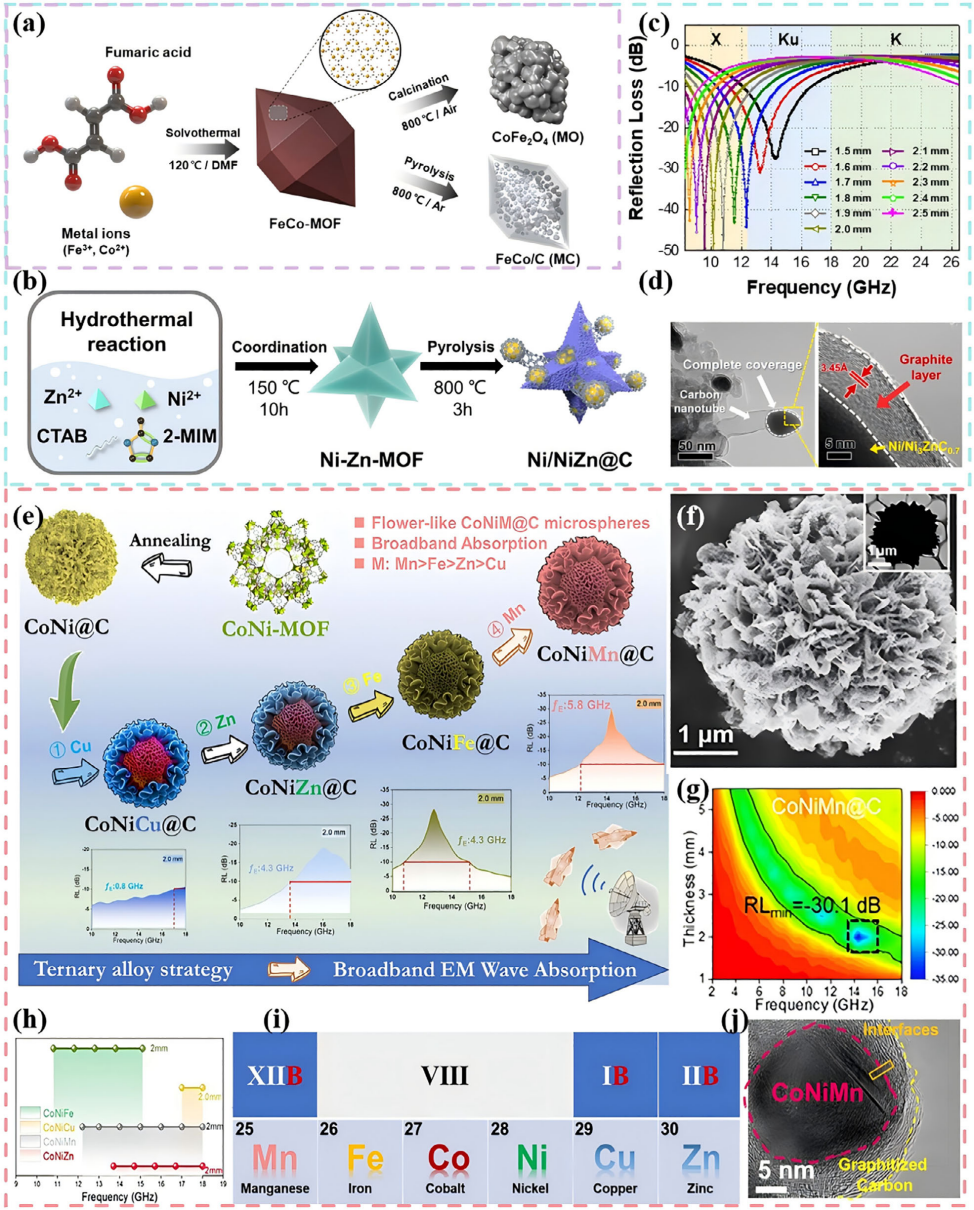
(a) Schematic of the synthesis process of FeCo‐MOF, MO, and MC [126] Copyright 2024, Springer, (b) 2D, 3D, and corresponding contour plots of MO_75_MC_25_ at different thicknesses, (c) Schematic of the preparation process of Ni/NiZn@C, (d) TEM and HRTEM images of S2 [127] Copyright 2023, Elsevier B.V., (e) design idea of broadband absorption in ternary MOFs‐derived CoNiM@C microspheres, (f) SEM images of CoNiMn@C, (g) reflection loss curves of CoNiMn@C, (h) EAB comparison of CoNiCu@C, CoNiZn@C, CoNiFe@C and CoNiMn@C samples, (i) positions of neighboring introduced elements in the periodic table, (j) HRTEM image of CoNiMn@C [128] Copyright 2024, Springer.

To further optimize impedance matching and attenuation, component modulation via multi‐metal synergism is essential. Compared to monometallic systems, bimetallic MOF derivatives offer superior tunability. Choi et al. [126]. derived two distinct phases from FeCo‐MOF (MIL‐88A): a conductive FeCo/C (MC) phase and a dielectric CoFe_2_O_4_ (MO) phase (Figure 13a). The oxide structure of MO regulated dielectric properties, while the porous MC phase provided abundant interfaces and defect sites. By adjusting the MC/MO ratio, they balanced conductivity and polarization; the optimized composite (30% MC) achieved an R_L_ of ‐52.29 dB and an EAB of 7.23 GHz. Similarly, Xie et al. [127]. synthesized NiZn‐MOF‐derived composites where metallic Ni/Ni_3_ZnCo_7_ particles were tightly wrapped by a graphitic carbon shell (Figure 13b). This core–shell structure optimized impedance matching through gradual changes in permittivity and extended wave paths via multiple scattering. Nitrogen doping further enhanced polarization losses, resulting in an *R*_L_ of ‐56.8 dB and an EAB of 5.9 GHz (Figure 13c,d). Introducing a third metal element allows for fine‐tuning of magneto‐crystalline anisotropy and saturation magnetization. Although bimetallic, MOF‐derived materials can enhance wave absorption through magnetic‐dielectric synergy, further breakthroughs are still needed to fine‐tune impedance matching and enhance broadband absorption capability. These breakthroughs are mainly attributed to component and structural homogeneity. For this reason, researchers have focused on ternary alloy systems to create multi‐component, heterogeneous interfaces by adding a third metal. Huang et al. [128]. developed flower‐like CoNiM@C microspheres (M = Mn, Zn, Cu, Fe) via a hot‐solvent method followed by calcination (Figure 13e,f). The CoNiMn@C variant exhibited the best performance, with an EAB of 5.8 GHz (covering 96.7% of the Ku band) at a thickness of 2.0 mm (Figure 13g,h). The ternary alloy core enhanced magnetic losses (natural resonance and eddy current loss), while the graphitized carbon shell and hierarchical porous structure facilitated conductive loss and multiple scattering (Figure 13i,j). This component modulation effectively balanced complex permittivity and permeability, ensuring efficient wave entry and dissipation.

Building upon the structural and compositional optimizations detailed in previous sections, the practical application of MOF‐derived hybrids hinges on overcoming synthesis‐induced limitations while leveraging their unique architectural advantages to achieve “thin, light, wide, and strong” performance. A primary bottleneck remains the reliance on high‐temperature pyrolysis (typically ≈800°C), which often triggers structural collapse, metal particle agglomeration, and uncontrolled graphitization, thereby degrading the microstructural features essential for wave absorption. To address these thermal instabilities, innovative strategies have been adopted from adjacent fields. Notably, Li et al. [129]. fabricated a novel Z‐type heterostructure by uniformly distributing CuS nanoflakes on Ti‐MOFs to form p‐n heterojunctions. This architecture not only prevents agglomeration and maintains a high specific surface area but also generates built‐in electric fields that promote charge separation, effectively stabilizing the active sites against thermal degradation. Further reinforcing structural integrity can be achieved by introducing robust protective layers or employing chemical bonding to regulate metal dispersion through in situ generation methods.

**FIGURE 14:**
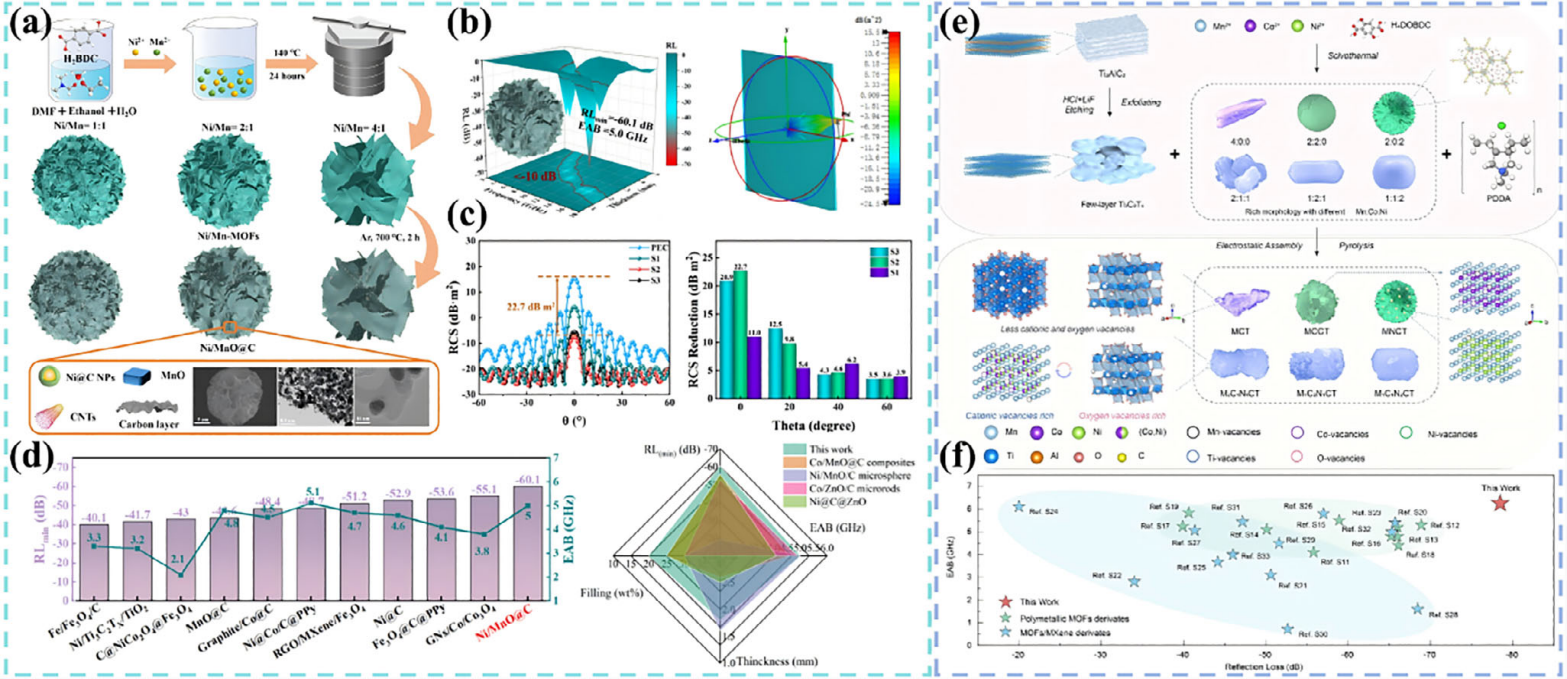
(a) Schematic illustration of the synthesis strategy for petal‐like lamellar Ni/MnO@C nanocomposites, (b) the 3D representations of *R*_L_ values and RCS simulation images for samples S2, (c) quantitative comparison of RCS reduction curve‐level reductions for S1, S2, S3, and PEC, (d) the comprehensive comparison of EMA performances between the present work and related literature [132] Copyright 2025, Elsevier B.V., (e) synthesis process and compositional characterization of MCNCT nanocomposites, (f) comparison of *R*_Lmin_ and EAB between polymetallic MOFs‐derived and MOFs/MXene‐derived nanocomposites [136] Copyright 2025, Elsevier B.V.

Beyond stability improvements, adjusting the composition and hierarchical structure of MOF‐derived materials has demonstrated significant potential for broadband wave absorption [111, 130, 131]. Xiang et al. [132]. synthesized Ni‐Mn‐O‐C nanocomposites with a unique, petal‐like, porous structure by creating heterogeneous interfaces [133, 134, 135]. They successfully integrated the ternary heterogeneous interface by scaling the Ni‐Mn metal‐organic framework (Figure 14a). The experimental results demonstrate that a 5.0 GHz absorption bandwidth can be achieved with a 1.8 mm material thickness while maintaining an efficient reflection loss of ‐60.1 dB (Figure 14b,c). This study aligns with the design principle of wide, thin, light, and strong for wave‐absorbing materials, offering insights into achieving a wide bandwidth, strong absorption, and a lightweight design with an ultra‐thin profile (Figure 14d). It's also worth noting that Hou et al. [136]. synthesized MnCoNi/MnTiO_3_@C@Ti_3_C_2_T_x_ nanocomposites using a combination of electrostatic self‐assembly and heat treatment (Figure 14e). The high concentration of oxygen vacancies and metal defects in M_2_C_1_N_1_CT results in a polarization loss that is more than one order of magnitude higher than the conductive‐loss contribution This achieves an effective absorption bandwidth of 6.25 GHz and a reflection loss of ‐78.47 dB. This is a significant improvement over most reported MOF‐based absorbers and represents progress in broadband absorption (Figure 14f).

MOF‐derived composite materials are designed by regulating the chemical properties and layered structures of metal ligands. Different combinations exhibit a variety of excellent properties. Extending the electromagnetic propagation path provides the material with the best impedance matching, achieving dielectric‐magnetic synergistic loss and perfect broadband efficient absorption.

Broadband absorption and attenuation strengths are further enhanced through core–shell design, multi‐metal alloying, and defect/interface engineering. However, while the material is close to being thin, light, broad, and strong, it must also overcome the challenges of thermal instability and amplification limitations during high‐temperature pyrolysis. Through step‐by‐step experimental exploration and continuous progress in heterostructure control, green synthesis, and integrated functions, MOF‐based absorbers will undergo a great transformation into electromagnetic applications.

Collectively, these advancements underscore that MOF‐derived composites, designed by regulating the chemical properties of metal nodes and organic ligands, can exhibit diverse and superior functionalities. Extending the electromagnetic propagation path through hierarchical porosity provides optimal impedance matching, thereby achieving synergistic dielectric‐magnetic losses and efficient broadband absorption While core–shell designs, multi‐metal alloying, and defect/interface engineering have further enhanced attenuation strengths, challenges regarding thermal instability during pyrolysis and scalability persist. Future efforts must therefore pivot towards refining pyrolysis kinetics and developing green, low‐temperature synthesis routes. Through continued progress in heterostructure control and integrated functional design, MOF‐based absorbers are poised to undergo a transformative shift from laboratory curiosities to practical solutions for next‐generation electromagnetic protection.

## 6 Conclusion and Outlook

EWAMs are considered as critical materials to solve electromagnetic interference problems in aerospace stealth, wearable electronics and high‐frequency communication systems. Electromagnetic wave‐absorbing materials are amongst the most promising functional materials. Improved dielectric loss synergism and enhanced broadband absorption can be realized with the adoption of multilevel porosity construction, particle modification, and multiscale interfacial network fabrication. In this review, the electromagnetic‐wave absorption mechanisms, dimensional engineering strategies, structural design principles, and recent research advances of CNF‐, MXene‐, and MOF‐derived absorbers are systematically summarized. Particular emphasis is placed on understanding how dimensional engineering and microstructure regulation influence dielectric/magnetic loss behavior, impedance matching characteristics, and broadband attenuation performance. By comparing representative dimensional engineering approaches and design strategies across different material systems, this review provides a comprehensive framework for the development of next‐generation lightweight and high‐efficiency EWAMs. Difficulties are encountered in balancing material performance, stability, and production scalability, especially under complicated synthetic routes. Table 1 provides a comparative overview of representative CNF‐, MXene‐, and MOF‐derived EWAM systems, highlighting their structural characteristics, attenuation mechanisms, and electromagnetic‐wave absorption performance.

**TABLE 1:** Summary based on multidimensional materials as EWAMs.

No.	Dimension	Morphology	Composition	*R*_L_ (dB)	EAB (GHz)	Thickness (mm)	Fillingratio (wt%)	Refs.
1	3D	Core–shell	MnCoNi/MnTiO_3_@C@Ti_3_C_2_T_x_	−78.47	6.25	2.1	30	[136]
2	2D	Porous core–shell nanosheets	CoFe_2_O_4_‐Co_3_Fe_7_@C	−119.7	9.2	1.8	50	[27]
3	0D	Core–shell	BST@ZnO/PVDF	−64.2	6.0	2.0	40	[28]
4	0D	Dandelion structures	SiO_2_@SiC@C	−64.2	6.0	2.0	20	[29]
5	3D	Magnetic chains	CC@Co/PDMS	−35.4	/	0.33	10	[137]
6	3D	Self‐rolling rod	Ti_3_C_2_T_x_@MoS_2_	−52.1	X‐Band	3.3	/	[138]
7	0D	Yolk–shell	CoNi@(SiO_2_or Air)@TiO_2_	−58.2	8.1	2.1‐2.5	/	[38]
8	3D	Magnetic coupling	CoNi PNPs	−59.9	6.82	1.8	/	[139]
9	3D	Closed porous	CF@Air@PMI	/	14.0	30	1‐4	[140]
10	3D	​MoC/Co@NC	Graphite/amorphous heterostructure	−47.72	4.58	2.5	15	[141]
11	3D	NCMF/MXene/CNF	Sandwich architecture	−65.1	9.4	2.8	N/A	[142]
12	0D	FeCoNi/Fe_7_Co_3_@C	Core–shell	−42.9	2.72	2.8	75	[143]
13	2D	Ti_3_C_2_T_x_@NiFe_2_O_4_	Non‐homogeneous interface	−43.9	5.78	4.0	30	[51]
14	3D	Co@C/MXene	Core–shell	−61.42	4.1	2.5	20	[52]
15	3D	NiO/Ni@CCS	Urchin‐shaped particles	−52.09	5.1	1.98	20	[53]
16	3D	Ho_2_O_3_@CNF	Cobweb	−61.37	6.88	2.64	25	[57]
17	1D	Co@CNF	Polyhedral frame encapsulation	−48.6	4.2	3.5	20	[144]
18	3D	CNA@FeCoNi MEA	Aerogel​	−56.7	5.28	2.0	5	[145]
19	1D	Fe_2_N@Ni_3_Fe/CNFs	Nanocube‐decorated nanofibers​	−56.75	6.7	2.3	/	[146]
20	1D	Co_3_Fe_7_@C	Hollow nanotubes	−107.4	11.6	1.4	5	[147]
21	3D	MCN@Fe_3_O_4_‐CF/PA6	Nanonet encapsulating magnetic nanoparticles	−65.3	6.76	1.8	/	[148]
22	3D	CSF‐FCIP2	Porous composite foam	−65.3	10.0	2.6	/	[149]
23	0D/1D/3D	Cu:CoSe_2_@CNT/CNF	Hierarchical core—shell	−68.4	7.11	2.23	15	[66]
24	1D	CoNi@C	Hollow porous carbon nanofibers	−45.9	5.92	1.49	15	[67]
25	3D	N‐doped RGO/CuFe_2_O_4_@PPy	Composite aerogels	−45.11	8.08	3.42	8	[69]
26	0D	Fe@SiO_2_@NC	Yolk‐shell spheres	−64.83	7.10	2.5	/	[150]
27	1D	PAN/Carbon	Hollow nanofibers	−44.73	6.60	1.76	30	[151]
28	0D	Fe, N‐codoped Carbon	Nanocages	−75.5	5.41	1.83	10	[152]

This review not only summarizes the state‐of‐the‐art developments of CNF‐, MXene‐, and MOF‐derived EWAMs, but also highlights the fundamental relationships between composition, microstructure, electromagnetic parameters, and absorption performance, thereby providing guidance for the rational design of next‐generation electromagnetic‐wave absorbers.

From the perspective of dimensional engineering, CNFs, MXenes, and MOF‐derived materials exhibit distinct structural characteristics and electromagnetic attenuation mechanisms, which collectively provide versatile pathways for optimizing EWAM performance. CNFs exhibit excellent dielectric‐loss capability owing to their high aspect ratio, interconnected conductive networks, and abundant polarization sites. Structural engineering strategies, including electrospinning, pore construction, and magnetic nanoparticle incorporation, have proven effective for enhancing impedance matching and dielectric‐magnetic synergistic attenuation. Magnetic nanoparticles are embedded in the fiber matrix, forming a unique structure that regulates heterostructures. Then, the dielectric constant is adjusted to optimize impedance matching. Meanwhile, surface defects generated by metal particle‐induced lattice distortion, together with the porous structure, provide the materials with dielectric‐magnetic synergistic loss benefits.

The abundant surface terminations on MXene nanosheets serve as polarization centers and facilitate interfacial charge accumulation, thereby enhancing dielectric polarization and electromagnetic energy dissipation. The atomically thin morphology and large specific surface area of MXenes provide abundant active sites for polarization and interfacial interactions, thereby enhancing electromagnetic attenuation capability. To overcome the impedance mismatch caused by the high conductivity of MXene, an optimized multi‐dimensional heterostructure combined with component doping is often used. The introduction of core–shell microspheres and hydrogen‐bonded interfacial networks effectively suppresses MXene restacking and promotes the formation of heterogeneous interfaces. The construction of multidimensional heterostructures and magnetic‐dielectric synergistic systems effectively improves both attenuation capability and effective absorption bandwidth.

The spatial confinement strategy of MOFs enables the uniform dispersion of dopant particles within carbon fibers. This allows the material to have a smaller filler volume while maintaining the same performance and meeting lightweight requirements. This strategy solves the problem of particle aggregation caused by high doping levels and prevents interfacial exfoliation resulting from insufficient surface‐active sites. The multi‐component composite system design optimizes the material's ratio, achieving a dynamic balance between impedance matching and attenuation capacity. These characteristics provide an effective pathway toward achieving the “thin, light, broadband, and strong” performance requirements of next‐generation EWAMs. Additionally, breakthroughs in the ternary alloy system and hierarchical structure optimize magnetic coupling strength by regulating magnetic crystal anisotropy, improving performance, and paving the way for further research.

The above results demonstrate significant improvements in the lightweight and broadband electromagnetic wave absorption performance. Despite the remarkable progress achieved thus far, several critical challenges must still be addressed before EWAMs can be widely implemented in practical applications.

1. To obtain a uniform pore structure for CNFs, the dispersion control technology of the template in the spinning solution must be thoroughly investigated to optimize the pore gradient distribution through spatial confinement.
2. CNFs have a high specific surface area and electrical conductivity, which allows them to excel in wave absorption. However, their 1D structure restricts the optimization of their electromagnetic wave propagation path.
3. MOF‐derived structures are at risk of collapsing structurally during the high‐temperature pyrolysis process. It is essential to achieve the right balance between stability and functionality, which can be done using molecular‐level coating technology and a low‐temperature gradient carbonization process.
4. MXene in a layered structure is prone to oxidative degradation in hot and humid environments, which increases costs and causes environmental problems.
5. During the electrostatic spinning process, unstable control of environmental temperature and humidity directly affects fiber morphology, size, and film‐forming properties. This, in turn, interferes with the homogeneity of the pore structure.
6. The vast, multivariable parameter space involved in tuning CNF‐, MXene‐, and MOF‐derived composites for optimal impedance‐matching and loss mechanisms makes manual trial‐and‐error optimization time‐consuming and prone to reproducibility issues. To advance further, there is a need to develop large, high‐quality datasets, standardize experimental protocols, and ensure seamless interoperability between AI models and diverse automation hardware.

Despite the remarkable electromagnetic‐wave absorption performance achieved under laboratory conditions, several critical bottlenecks continue to hinder the practical deployment of EWAMs. Long‐term environmental stability remains insufficiently understood, particularly under conditions involving humidity, temperature fluctuations, and mechanical stress. Many high‐performance EWAMs rely on complex multistep synthesis routes, which increase production costs and limit scalability. Simultaneously achieving lightweight design, ultrathin thickness, broad effective absorption bandwidth, and strong attenuation remains challenging for practical applications such as aerospace stealth and wearable electronics. Finally, the lack of standardized testing protocols and large‐scale, high‐quality databases hinders reliable performance comparison and slows the development of data‐driven material design strategies.

Beyond the scientific challenges discussed above, several key engineering and commercialization bottlenecks must also be addressed before CNF‐, MXene‐, and MOF‐derived EWAMs can be translated from laboratory‐scale demonstrations to practical applications.

1. The practical applications of CNFs are restricted due to their limited and weak effectiveness in the low‐frequency band. Designing a heterogeneous structure can strengthen the multi‐frequency response and form a magneto‐dielectric synergistic system that optimizes impedance‐matching characteristics. Furthermore, a broad frequency spectrum is the target of magnetocrystalline anisotropy modulation through a ternary alloy system.
2. MXene is susceptible to oxidative degradation in hot, humid environments, while MOF‐derived materials risk structural collapse during carbonization at high temperatures. Molecular‐level coating technology and a low‐temperature gradient carbonization process can solve interfacial stability issues and inhibit pyrolytic degradation.
3. For aerospace and portable electronic applications, a persistent challenge lies in simultaneously achieving lightweight design, ultrathin thickness, and strong attenuation performance. Excessive thickness affects the application of materials in various ways. Designing a gradient multilayer structure can reduce the matching thickness by utilizing the phase offset mechanism of the surface layer. Through dielectric adjustment of the sheath layer, we expect to achieve broadband absorption at an ultra‐thin thickness.
4. The strong acid etching process used to produce MXene creates harmful byproducts, and it is difficult to eliminate all volatile substances during preparation. These substances can cause chronic harm to the human body over time. Developing green etching alternatives and a resource recycling system can reduce environmental risks and the cost of developing practical applications. However, the green process still needs further exploration.
5. Integrating AI‐driven, multi‐objective optimization algorithms with closed‐loop automated synthesis and characterization platforms offers a promising route toward accelerated material discovery. However, the effectiveness of such approaches is currently limited by the lack of standardized, high‐quality experimental databases and interoperable automation platforms. This process pinpoints high‐performance formulations and processing conditions for electromagnetic wave absorption.

**FIGURE 15:**
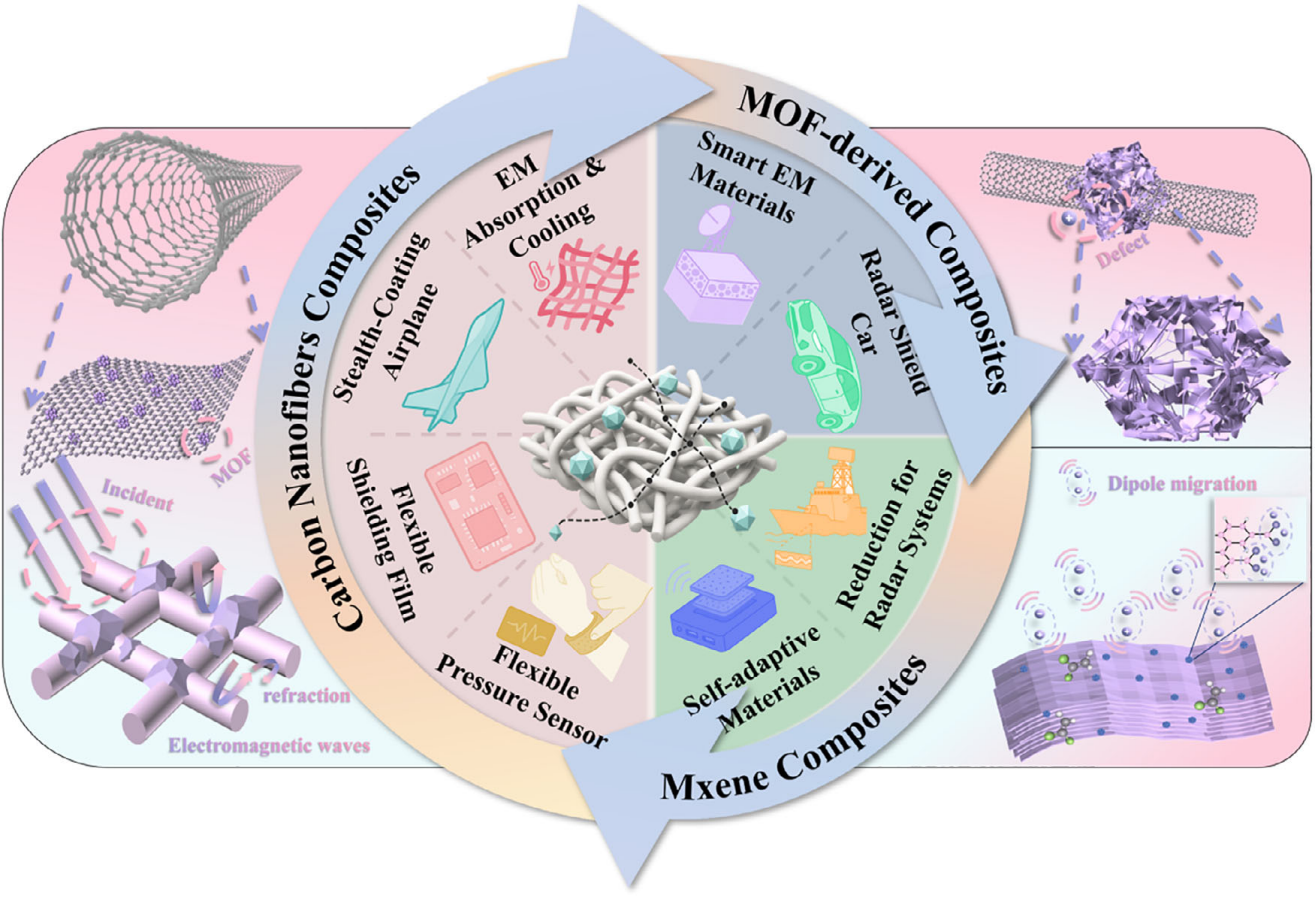
Future prospects of MXenes, CNFs and MOFs.

Although significant advances have been achieved through the optimization of individual material systems, future breakthroughs will increasingly depend on the development of multifunctional and synergistic composite architectures. The current environment requires multifunctional and adaptive products to overcome bottlenecks. Hybrid composites integrating 1D, 2D, and 3D structural features offer unique opportunities to exploit dimensional engineering for synergistic enhancement of electromagnetic‐wave absorption performance. Accordingly, substantial opportunities remain for the continued development of high‐performance electromagnetic‐wave absorbing materials (Figure 15). Addressing emerging scientific and engineering challenges will further accelerate progress in this field. The complementary advantages of CNFs, MXenes, and MOF‐derived materials provide valuable opportunities for overcoming the limitations of individual material systems. In addition, it remains challenging to achieve a balance between broadband absorption, ultra‐thin thickness, lightweight design, and strong attenuation. Future research should focus on the rational design of stable material architectures, scalable and environmentally friendly manufacturing processes, standardized performance evaluation protocols, and data‐driven optimization strategies. Combining structure–function mechanisms with processing control is also crucial to realizing next‐generation wave‐absorbing materials for everyday applications in aerospace, stealth, and wearable electromagnetic management.

## Author Contributions

**Chuanyu Liu**: data curation, formal analysis, and Writing – original draft. **Xiaoxin Guo**: data curation and formal analysis. **Xunlong Zhang**: conceptualization, investigation, methodology, and writing – review & editing. **Ruiling Sun**: data curation and formal analysis. **Xingyu Zhang**: data curation and formal analysis. **Can Ge**: investigation and conceptualization. **Xuhong Yang**: data curation and formal analysis. **Yuqing Liu**: supervision, validation, methodology, and writing – review & editing.

## Conflicts of Interest

The authors declare no conflicts of interest.

## Supporting information




**Supporting File**: advs77294‐sup‐0001‐SuppMat.docx.

## Data Availability

The data supporting this study's findings are available from the corresponding author upon reasonable request.
