# Supplementary material for: Dimensional Engineering of Carbon‐Based Hybrids: From 1D Conductive Networks to 3D Porous Architectures for Next‐Generation Microwave Absorption
Source: Adv Sci (Weinh). 2026 Aug 20;13(52):e77294. doi: 10.1002/advs.77294 (PMC13491372; doi:10.1002/advs.77294)
Supplement: Supplementary file 1 [file ADVS-13-e77294-s001.docx]

Chuanyu Liu is currently pursuing a degree in Textile Engineering at Soochow University. Since 2024, her current research has focused on the application of carbon nanofiber materials in flexible electrodes, electromagnetic wave absorption, and supercapacitors.

Xiaoxin Guo is currently pursuing a degree in Textile Engineering at Soochow University. Since 2024, his research direction has focused on the application of carbon nanofibers in fiber electrodes and piezoelectric materials.

Xunlong Zhang is a young scholar at the School of Polymer Science and Engineering, SiChuan University, China. His research focuses on carbon nanofiber, electrochemistry, flexible energy storage materials, porous materials.

Can Ge is a professor at department of Electronic Engineering and Shun Hing Institute of Advanced Engineering. His current research interests focus on the synergy of fibrous materials in solar-heat-water-electricity.

Xuhong Yang is currently a professor at Soochow University in China. Her main research directions include the recycling and utilization of keratin and the development of functional materials

Yuqing Liu is currently a professor at Soochow University in China. His research interests include nano functional fibers, photoelectrocatalytic fiber materials, and new energy fiber materials.
